# Routine milk records reveal novel two-way interactions shaping ketosis risk in German dairy cows

**DOI:** 10.1371/journal.pone.0353380

**Published:** 2026-07-16

**Authors:** Franziska Gheronte, Martina Hoedemaker, Kerstin-Elisabeth Müller, Gabriela Knubben-Schweizer, Roswitha Merle, Dörte Döpfer, Nora Mansfeld, Yury Zablotski

**Affiliations:** 1 Clinic for Ruminants with Ambulatory and Herd Health Services, Ludwig-Maximilians Universität Munich, Oberschleissheim, Germany; 2 Clinic for Cattle, University of Veterinary Medicine Hannover Foundation, Hannover, Germany; 3 Clinic for Ruminants and Swine, Freie Universität Berlin, Berlin, Germany; 4 Institute of Veterinary Epidemiology and Biostatistics, Freie Universität Berlin, Berlin, Germany; 5 Food Animal Production Medicine, School of Veterinary Medicine, Madison, Wisconsin, United States of America; Eskisehir Osmangazi University: Eskisehir Osmangazi Universitesi, TÜRKIYE

## Abstract

Ketosis is one of the most prevalent metabolic disorders in dairy herds, with significant economic and welfare implications. However, existing literature often examines risk predictors in isolation, and their interactions and dependencies remain poorly understood. This study investigated how production, health, and management factors – and their interactions – are associated with ketosis risk (KR) at the animal level in German dairy cows, with a focus on German Holstein (GH) and German Simmental (SIM) breeds. Data from 76,809 cows across 717 German dairy farms collected between 2015 and 2019 were analyzed using multivariable generalized linear mixed-effects logistic regression with two-way interactions and random forest algorithms. KR was predicted using a non-invasive, widely applicable approach based on milk composition thresholds derived from monthly test-day records. Key risk factors included breed, parity, energy-corrected milk (ECM) yield, body condition, lameness, housing, and lactation stage. Twelve significant two-way interactions were identified, highlighting the multifactorial and interdependent nature of KR, most frequently involving lactation stage (six interactions), followed by ECM yield (five interactions) and breed (four interactions). The overall predicted probability of KR was 7.1%. Although high-yielding multiparous cows showed the highest absolute KR (prob. = 13.6% [95% CI 12.5–14.8]), high-yielding primiparous cows exhibited an unexpectedly elevated KR (prob. = 11.4% [95% CI 9.9–13.1]). Elevated KR was also observed in underconditioned cows during mid-early lactation (days in milk [DIM] 30–99, prob. = 21.0% [95% CI 18.6–23.5]), and SIM cows producing high ECM yields (>35 kg/day; prob. = 19.5% [95% CI 16.2–23.4]). The highest predicted KR occurred in underconditioned, first-parity cows housed in tie stalls, lame, and producing high ECM yields during mid-early lactation, indicating that specific combinations of risk factors amplify KR beyond the sum of their individual contributions. These findings highlight the importance of modeling interactions among animal-level and environmental factors to enable context-specific risk assessment. Evaluating breed, lactation stage, body condition, production level, housing, and health status jointly may support more targeted prevention strategies, particularly during the first 99 DIM, and improve identification of cows at elevated ketosis risk.

## Introduction

Ketosis is a common metabolic disorder of early-lactation dairy cows. During the transition period from late pregnancy to early lactation, cows typically enter a pronounced negative energy balance (NEB) as milk production rises faster than dry matter intake (DMI) [1]. This NEB triggers the mobilization of body reserves including muscle and fat tissues, potentially culminating in subclinical or clinical ketosis. Subclinical ketosis (SCK) occurs without obvious clinical signs but has substantial economic and welfare consequences [2,3]. SCK is prevalent in a significant proportion of cows, with an estimated global prevalence above 20% [4], and has been reported to be particularly high in Western European herds (e.g., Germany, France, The Netherlands) [1,5]. Cows with SCK produce less milk than expected, have poorer fertility, and are more susceptible to disease, e.g., with elevated odds of metritis, mastitis, or displaced abomasum [6–10]. From a pathophysiological perspective, Zhang and Ametaj (2020) [10] proposed a classification scheme distinguishing the types of ketosis. Type I typically occurs between 3 and 6 weeks postpartum and is characterized by chronic hypoglycemia driven by high milk production; it therefore reflects a state of NEB. Type II ketosis occurs immediately postpartum and is associated with overconditioning during the dry period, whereby excessive mobilization of body fat drives hepatic triglyceride accumulation and impaired gluconeogenesis. Type III ketosis is attributed to dietary factors, specifically the ingestion of feed with high butyric acid content caused by *Clostridium* spp. contamination in improperly fermented silage.

Given these productivity and health consequences, ketosis is a critical concern in the dairy industry. In Germany, where dairy farming is a vital sector, understanding and mitigating the predictors for ketosis risk (KR) is essential for maintaining the productivity and sustainability of farms. Reports on the prevalence of ketosis show significant differences between breeds and management systems [2,6,9,11], underscoring the need for targeted research to address breed-specific susceptibilities. This is particularly relevant in German dairy herds, where high-yielding Holstein-Friesians and dual-purpose Simmental cows are typically kept under diverse conditions that vary by geographic region (e.g., mountainous vs. flatland areas), housing type (tie-stall vs. free-stall housing), and health status (e.g., presence of lameness or poor body condition). Moreover, concentrating on risk factor assessment using non-invasive indicators may allow for intervention prior to clinical disease onset. This proactive approach may not only mitigate adverse economic and health outcomes but also fundamentally enhance animal welfare by addressing metabolic imbalances in their subclinical or early stages.

Previous studies have identified several cow-level predictors of ketosis. Parity and body condition are consistently implicated: multiparous cows and those calving with high body condition scores (BCS) face markedly higher risk of ketosis [12,13]. High-yielding dairy cows (energy corrected milk (ECM) yield > 35 kg in our study) tend to have elevated milk fat and reduced protein in early lactation [14]. Moreover, a raised test-day milk fat-to-protein ratio (FPR, often > 1.4) is a classic indicator of SCK [15–17]. Thus, the elevations in test-day FPR, particularly in high-producing cows, are interpreted as indicative of increased KR. Other factors include seasonal effects and milk yield in previous lactations: cows calving in early spring or summer, those subjected to long dry periods (>60 days), or cows with a long-lasting period of high milk yield in the previous lactation have shown increased ketosis incidence in previous studies [13,18]. Despite these findings, significant knowledge gaps persist. Although breed has long been identified as an influential risk factor for ketosis [19], breed-specific risk profiles are poorly analyzed, particularly in multi-breed farms. Some breed differences were reported; e.g., higher ketosis prevalence was observed in Jersey cows compared to Holsteins [1]. However, most field surveys focus on Holstein-Frisians (HF), leaving the Simmental (SIM) largely unstudied. Moreover, despite Germany’s seamless system of milk-recording data and recording of periparturient events offering a unique opportunity to study thousands of cows across regions, few analyses have leveraged such large-scale national datasets to model KR.

Most studies have applied statistical models (logistic or mixed-effects logistic regression) to relate predictors, such as milk-record data (fat, protein, yield), days in milk (DIM) etc., to blood levels of ketone bodies [13,15]. For instance, Vanholder et al. (2015) used a mixed-effects logistic model (accounting for herd) to quantify how parity, BCS, and milk components predict ketosis. In sum, the literature highlights parity, metabolic status (e.g., BCS, FPR), lactation performance, and season as key risk factors for ketosis.

In addition, most studies examine predictors solely as main factors via multivariable models, overlooking the fact that their associations can vary considerably depending on other concurrent conditions. Thus, detailed analyses of how major risk factors interact are scarce. For instance, the combined dynamics of production level and management system, or of health status and lactation stage, are severely understudied. This also applies to lactation stage-dependent variations: while early lactation is widely acknowledged as a high-risk period, the possibility that the same risk factor may behave markedly differently in early lactation (DIM 0–29) compared with mid-early lactation (DIM 30–99) has received little systematic attention.

Addressing these gaps is essential for identifying the precise timing and context in which individual risk factors exert their strongest association with KR, and for understanding how concurrent variables (e.g., housing, lameness, breed, body condition) modify these relationships. Such knowledge will help define specific high-risk profiles and guide targeted interventions. Beyond this, we introduce a novel, non-invasive approach for quantifying KR suitable for large-scale implementation using routinely collected milk-record data to detect energy lack via FPR (cut-off value at 1.4 [20]). Multiple studies demonstrate that elevated FPR is an indicator of (subclinical) ketosis and KR [15–17]. While blood-based metabolite measurements constitute the diagnostic gold standard for confirming clinical ketosis, they are often impractical for continuous, large-scale monitoring of individual animals. In contrast, our approach shifts the focus from direct disease diagnosis to the early identification of metabolic imbalances at the individual animal level. By targeting the underlying risk, this method may enable the detection of vulnerable animals approaching NEB, thereby facilitating preventive decision-making prior to the onset of overt metabolic dysfunction.

This study, therefore, aims to quantify not only animal-level predictors for KR in German dairy cows, using a large national cross-sectional dataset, but also to explore the two-way associations among these. We model KR (based on milk-record data) as a function of lactation stage, ECM yield, breed, parity, body condition, housing and lameness. Advanced statistical approaches are applied, including generalized linear mixed logistic regression (to account for farm-level clustering) and random forest algorithms (to assess variable importance). By identifying general risk factors as well as key two-way interaction patterns, this work supports more precise, context-specific herd management and aligns with recent calls for tailored, geography-specific ketosis mitigation strategies [21].

## Materials & methods

### Farm recruitment and data collection

In a cross-sectional study on animal health, biosecurity, and housing circumstances, dairy farms from three different regions of Germany were randomly chosen, namely the Region North (Lower Saxony and Schleswig-Holstein), Region East (Saxony-Anhalt, Brandenburg, Mecklenburg-Western Pomerania, and Thuringia), and Region South (Bavaria) [22,23]. Data from the national animal information database (HIT) and farm data from the Milchprüfring Bayern e.V. were used for reference. To determine the ideal and practical sample size, various scenarios were computed using a power of 80% and a 5% level of significance. Additionally, cut-off values for farm size specific to each region were established to ensure that the sample population had a realistic distribution of farm sizes and to account for structural differences in dairy farming in different regions of Germany [24]. A total of 253 farms from the North, 252 from the East, and 260 from the South were included in the study, all farmers had received an invitation with information given on the study by mail and voluntarily chose to participate. Each farm was to be visited on a single occasion.

The University Animal Welfare Representative at the University of Veterinary Medicine Hannover (Germany) was consulted and confirmed that formal ethical review and approval were not required for this study. At the time the research was planned and conducted, prospective approval by an animal or human research ethics committee was not mandated under German regulations [25,26]. Data collection was conducted on the farms between December 2016 and August 2019 while maintaining the anonymity of the participating farms, in accordance with German and European data protection laws. After receiving written consent from the contributing farms, the data on parity, DIM, breed, milk yield, milk constituents (milk fat, milk protein, and somatic cell count (SCC)) were obtained from the national milk recording system (DHI). Production data, including milk yield and composition, were there gathered once monthly – and were at hand up to 12 months before the farm visit – during a test day sampling. Each cow underwent an individual scoring procedure. If the herd size exceeded 130 cows, a maximum of 130 cows were randomly scored. The scoring process ensured that there was an even representation of cows at the feeding area, cows resting, standing, and being in motion. Cows that were scored and those that were not, were marked with two different livestock marking sprays for identification purposes. The number of unassessed cows was counted to determine the correct number of cows present. During the farm visit, all animals that were lactating or at dry-off and housed in tie stalls were individually evaluated for body condition and lameness. The researchers conducted live stall lameness scoring to document weight shifting between feet, standing on one foot while sparing the other, uneven weight bearing when stepping from side to side, and standing on the edge of the kerb [27]. They examined each cow for 90 seconds from both the rear and a caudolateral perspective. If at least two of the four aforementioned indicators were present, the cow was considered lame [27,28]. Cows on pasture during the visit were not scored for lameness. To assess body condition, the researchers used a five-point scale ranging from 1 to 5, with 0.25 increments [29,30].

One crucial aspect to consider when analyzing the provided data is that all gathered information reflects a single point in time. As such, there is no available information on the animals’ health history prior to this point. As a result, all risk factors are assessed only in relation to the moment when the respective cow was evaluated, the KR was determined at this one particular moment in time using our calculation (further explained in the “Data handling” section below).

### Data handling

The data were cleaned by filtering for lactating cows, removing invalid values (e.g., −66), standardizing date formats, and removing duplicates. Milking and visit data were merged using a 100-day time window around the visit day to capture relevant milking records while accounting for temporal variability. Within the 100-day window, up to four milk-record observations per cow were aggregated using a Bayesian bootstrap (1,000 iterations; *bayesboot* package in R) to compute weighted means for milk yield, fat percentage, protein percentage, DIM, and BCS. Aggregation was performed at the level of farm, cow, visit date, and breed, thereby aligning milk-record data with the corresponding on-farm assessment visit. Few missing values were imputed using the missRanger R package, which used a random forest-based algorithm (with 1000 trees and a fixed starting value for the random number generator to secure the reproducibility). In the first stage, missing values for milk metabolites, BCS, and DIM were imputed at the individual animal level. The proportion of missing values was very low: 3.2% for lameness status, 1.8% for lactation stage, and <0.5% for BCS and milk metabolites (urea, somatic cells).

In the second stage, after aggregating data and merging with additional farm-level information as well as the prescribed bootstrapping, a final imputation was performed to account for missing values in parity (3.37%) as well as lameness, breed and condition (all < 0.01%). Housing data were completely intact. All available animal- and farm-level variables were used as predictors in the Random Forest algorithm to ensure the highest possible imputation accuracy.

Crucially, the target variables (FPR and KR) were mathematically derived only after the final imputation stage was completed. This sequential approach was chosen to maintain the maximum number of observations while strictly avoiding any circular reasoning or bias in the estimation of the metabolic outcome.

Due to a small sample size for most breeds, all breeds other than German Holstein (GH) and German Simmental (SIM) were combined into a “mixed” category. BCS was rounded to the nearest quarter-point to align with standard scoring conventions. The scores then were categorized according to what has been referred to for different breeds and lactation stages as underconditioned, optimally conditioned (“normal”), or overconditioned [31–35], as has been established by PraeRi (2020) [22]. DIM were categorized into lactation stages to simplify the analysis. The following scores were used to classify the days postpartum (p.p.): early lactation = 0–29 days p.p.; mid-early lactation = 30–99 days p.p.; mid-late lactation = 100–199 days p.p.; late lactation = 200–299 days p.p., dry = < 0 & > 299 days p.p. [36]. Lameness was assessed by combining limb locomotion and stall lameness scores into a single categorical variable (“lame” or “not lame”), retaining only cows with consistent lameness scores. Parity was systematized into three groups: primiparous cows (parity 1), second-parity cows (parity 2), and multiparous cows (parity 3+). Cows were moreover grouped by their housing as either “loose” (free-stall housing) or “restrictive” (tie-stall housing).

The response variable “ketosis risk” was categorized into “yes” or “no”. To classify whether a cow had risk for ketosis, the milking data were processed in several steps, as the association between FPR and metabolic vulnerability is consistently confirmed [16]. Following recent recommendations by Glatz-Hoppe et al. (2019), we implemented multiple threshold values concerning milk yield, milk protein, and milk fat contents [37]. By evaluating milking data with individual upper and lower limits for milk fat and milk protein per cow, contingent upon milk yield (in kg), which demonstrates an inverse correlation with milk protein and milk fat content [37], the metabolic status of dairy cows can be estimated with significantly greater accuracy. We computed four dynamic limits for milk protein and fat per cow (Fat_max_, Fat_min_, Protein_max_, Protein_min_) instead of relying on the static limits previously proposed [38]. The FPR was then calculated and categorized as “normal” (FPR ≤ 1.4) or indicative of a “risk of ketotic metabolic status” (FPR > 1.4), serving as a non-invasive proxy for metabolic imbalance. As described by Glatz-Hoppe et al. (2019), this risk of ketotic metabolic status can be more accurately identified by a FPR > 1.4 combined with either a milk fat content > Fat_max_ or a milk protein content < Protein_min_ [37].


$$\begin{array}{c} E_{min}=(\text{4},\text{1}\text{1}-\text{0},\text{0}\text{2}\text{3}\times kg\;milk/day)\times (\text{1}-\text{0},\text{3}\text{5}/\text{3},\text{5}\text{1}) \end{array}$$

$$\begin{array}{c} F_{max}\;=(\text{5},\text{0}\text{6}-\text{0},\text{0}\text{3}\text{3}\times kg\;milk/day)\times (\text{1}+\text{0},\text{6}\text{8}/\text{4},\text{2}\text{0}) \end{array}$$

For better comparison of lactation performance and global milk production, we used ECM yield instead of the usual daily milk yield, as it considers the individual composition of each cow’s milk and its energetic value [39]. ECM yield was calculated using the following formula [40,41]:


$$\begin{array}{c} \text{ECM yield}=\frac{milk\;yield(kg)\times \left[\text{0},\text{3}\text{8}\times \left(milk\;fat(\%)\right)+\text{0},\text{2}\text{1}\times \left(milk\;protein(\%)\right)+\text{1},\text{0}\text{5}\right]}{\text{3},\text{2}\text{8}} \end{array}$$

ECM yield was subsequently categorized into three groups: < 20 kg per day for cows with low milk yield, 20–35 kg per day for cows with average milk yield, and > 35 kg per day for cows with high milk yield.

### Statistical analysis

Both data handling and all statistical analyses were performed using the open-source R statistical software (R: The R Project for Statistical Computing [42]) with the RStudio Desktop interface [43]. A comprehensive list of all R packages and their respective versions used in this study is provided in S1 File. The statistical analysis was conducted at the individual animal level. All given data underwent univariable analysis to determine the association of each predictor with the target variable, “ketosis risk”, using generalized linear mixed-effects logistic regression with a random effect of farm on the intercept.

A multi-stage selection strategy was employed: initial candidate variables were selected a priori based on clinical relevance. Subsequently, univariable generalized linear mixed-effect models (GLMMs) were employed, where the individual association of each variable was evaluated using Type II Wald chi-square tests (via the car::Anova package). All predictors that scored a p-value of less than or equal to (≤) 0.05 were considered for further multivariable analysis. This ensured that only the most biologically and statistically relevant predictors were retained. Some variables have been excluded from the subsequent multivariable model due to multicollinearity issues (e.g., ECM yield is highly correlated with milk fat, milk protein, and milk yield). The final multivariable model studied KR through the fixed effects of breed, ECM yield, condition, lactation stage, lameness, parity, and housing by comparing their categories. The importance of predictors on the response variable KR was evaluated by (1) using analysis-of-variance with chi-square tests and (2) a random forest algorithm.

To further evaluate the associations of all factors with KR, all possible pairwise two-way interactions between the seven main predictors were systematically evaluated. For each combination, a separate GLMM including the two respective main effects, their interaction term, and the random intercept of farm was constructed. The significance of each interaction was evaluated using Type II Wald chi-square tests. All significant interactions with a p-value less than (<) 0.05 were summarized in the final graph. To account for multiple comparisons during post-hoc analysis of these interactions, p-values were adjusted using the Benjamini-Hochberg (BH) method to control the false discovery rate (FDR). Detailed results of all contrasts and pairwise comparisons can be found in S2 File.

## Results

### Descriptive statistics of a dataset

This study included 76,809 dairy cows from 717 farms across Germany. Cows were categorized by breed as follows: 62,759 Holstein Friesian (81.7%), 8,228 Simmental (10.7%), and 5,822 cows of other breeds (7.6%). Parity distribution showed 26,625 first parity cows (34.7%), 19,916 cows in their second parity (25.9%), and 30,268 cows in parity 3 or higher (39.4%). Of the total population, 5,443 cows had KR (7.1%), while 71,366 cows displayed no KR (92.9%). Lactation stage was assessed using DIM: 4,222 cows (5.5%) were in early lactation (DIM 0–29), 14,402 (18.7%) in mid-early lactation (DIM 30–99), 20,766 (27.0%) in mid-late lactation (DIM 100–199), and 19,575 (25.5%) in late lactation (DIM 200–299). Additionally, 17,844 cows (23.2%) were in the dry period (DIM < 0 or >299). Housing conditions indicated that 74,871 cows (97.5%) had free-stall housing (loose), while 1,938 (2.5%) were kept in tie-stall housing (restrictive). Lameness was observed in 27,558 cows (35.9%), with 49,251 (64.1%) classified as not lame. Body condition assessment revealed 47,509 cows (61.9%) in normal condition, 18,944 (24.7%) overconditioned, and 10,356 (13.5%) underconditioned. Milk yield, measured as ECM yield, showed 8,867 cows (11.5%) as low-yielding (ECM yield <20 kg), 48,715 (63.4%) as average-yielding (ECM yield 20–35 kg), and 19,227 (25.0%) as high-yielding (ECM yield >35 kg).

The descriptive statistics of the data set, split into cows with ketosis risk and cows without, are displayed in Table 1.

**Table 1:** Descriptive statistics of potential predictors for ketosis risk in dairy cows by ketotic risk status.

Predictor	KR N = 5,443^1^	no KR N = 71,366^1^
**Breed**		
*GH*	4,179 (6.7%)	58,580 (93%)
*SIM*	746 (9.1%)	7,482 (91%)
*Mixed*	539 (9.3%)	5,283 (91%)
**Lactation stage**		
*0-29*	578 (14%)	3,644 (86%)
*30-99*	2,361 (16%)	12,041 (84%)
*100-199*	1,027 (4.9%)	19,736 (95%)
*200-299*	700 (3.6%)	18,982 (96%)
*Dry*	798 (4.5%)	16,942 (96%)
**Condition**		
*Normal*	3,461 (7.3%)	44,051 (93%)
*Overconditioned*	1,073 (5.7%)	17,867 (94%)
*Underconditioned*	930 (9.0%)	9,427 (91%)
**Parity**		
*1*	1,678 (6.3%)	24,943 (94%)
*2*	1,203 (6.0%)	18,721 (94%)
*3+*	2,583 (8.5%)	27,681 (91%)
**Housing**		
*Loose*	5,254 (7.0%)	69,617 (93%)
*Restrictive*	210 (11%)	1,728 (89%)
**Milk yield**	28 (21, 36)	29 (23, 35)
**Milk fat**	5.06 (4.63, 5.53)	4.03 (3.65, 4.44)
**Milk protein**	3.27 (3.03, 3.61)	3.48 (3.26, 3.73)
**ECM yield categories**		
*< 20*	698 (7.9%)	8,169 (92%)
*20-35*	2,662 (5.5%)	46,043 (95%)
*> 35*	2,104 (11%)	17,133 (89%)
**Lameness**		
*Lame*	2,130 (7.7%)	25,498 (92%)
*Not lame*	3,334 (6.8%)	45,847 (93%)
**Fat-to-protein ratio**	1.50 (1.44, 1.59)	1.16 (1.07, 1.25)

^1^n (%); Median (Q1, Q3).

Table 1 (N = 76,809) compares cows with ketosis risk (KR, n = 5,464) to those without (n = 71,345). Categorical variables are reported as frequencies and percentages, while continuous variables are summarized using medians and interquartile ranges (Q1, Q3) to describe central tendency and variability.

### Variable importance

**Fig 1:**
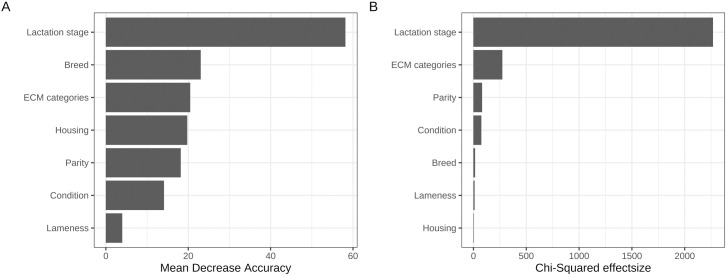
Comparison of variable importance for predictors of ketosis risk. Importance was assessed using **(A)** the Random Forest classification algorithm and **(B)** a multivariable mixed-effects logistic regression model (N = 76,809). Panel A displays the Mean Decrease Accuracy from the Random Forest, representing the loss in model prediction performance when a variable is permuted. Panel B ranks the risk factors by their importance based on Type II Wald Chi-Square values from an analysis of deviance. In both panels, higher values indicate stronger relative associations or higher importance for predicting ketosis risk (KR).

Fig 1 illustrates the relative importance of predictors for KR using two statistical methods. Panel A presents the Mean Decrease Accuracy from a Random Forest classification algorithm, which identifies lactation stage as the most influential predictor (score = 58.17), followed by breed (score = 23.02) and ECM yield categories (score = 20.46). Housing (score = 19.78) and parity (score = 18.20) rank fourth and fifth, respectively, while body condition (score = 14.14) and lameness (score = 4.00) are the least influential predictors according to this method.

Panel B displays the Chi-Squared effect sizes from Type II Wald Chi-Square tests in a multivariable mixed-effects logistic regression. Lactation stage again emerges as the most critical risk factor (Chi-Squared = 2240, p < 0.001), followed by ECM yield categories (Chi-Squared = 274, p < 0.001). The statistical contribution of ECM yield was approximately one-tenth of that observed for lactation stage. Parity (Chi-Squared = 83, p < 0.001) and body condition (Chi-Squared = 75, p < 0.001) exhibit similar effect sizes, while breed (Chi-Squared = 16, p < 0.001) and lameness (Chi-Squared = 11, p = 0.001) are less influential. Housing has the smallest effect size (Chi-Squared = 5, p = 0.024), indicating the least importance among the predictors in this analysis.

### Univariable and multivariable results

**Fig 2:**
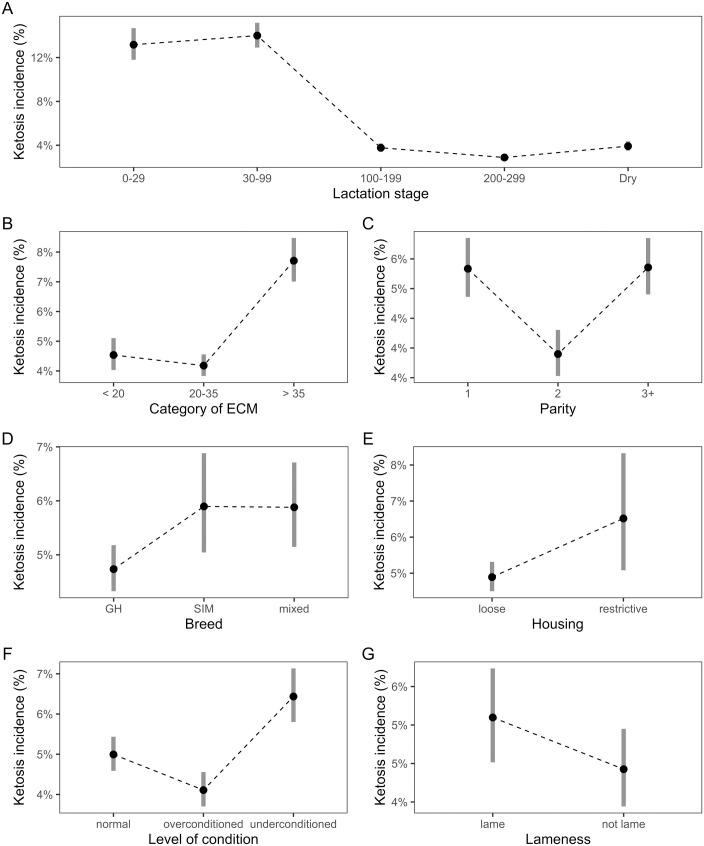
Predicted probabilities of ketosis risk across key predictors based on multivariable mixed-effects logistic regression. The plot illustrates the estimated probability of ketosis risk (KR) across different levels of the identified risk factors (N = 76,809). Points show the mean predicted probability, while shaded areas and error bars represent the 95% confidence intervals. Probabilities are adjusted for all other predictors in the model and account for the random effect of the farm. Panels display: **(A)** lactation stage, **(B)** energy-corrected milk yield (ECM yield, kg), **(C)** parity, **(D)** breed, **(E)** housing, **(F)** body condition, and **(G)** lameness.

Fig 2 provides an overview of the predicted probabilities of ketosis estimated by the multivariable mixed-effect logistic regression for all included risk factors, while univariable and multivariable results are compared in Table 2.

**Table 2:** Odds ratios, 95% confidence intervals, and p-values for cow-level predictors of ketosis risk from univariable and multivariable mixed-effects logistic regressions.

	Univariable			Multivariable		
Predictor	OR^1^	95% CI	p-value	OR^1^	95% CI	p-value
**Lactation stage**						
*(30-99)/ (0-29)*	1.30***	1.12, 1.50	**<0.001**	1.07	0.92, 1.25	0.729
*(100-199)/ (0-29)*	0.30***	0.26, 0.35	**<0.001**	0.26***	0.22, 0.31	**<0.001**
*(100-199)/ (30-99)*	0.23***	0.21, 0.26	**<0.001**	0.24***	0.21, 0.27	**<0.001**
*(200-299)/ (0-29)*	0.20***	0.17, 0.24	**<0.001**	0.20***	0.16, 0.23	**<0.001**
*(200-299)/ (30-99)*	0.16***	0.14, 0.18	**<0.001**	0.18***	0.16, 0.21	**<0.001**
*(200-299)/ (100-199)*	0.68***	0.59, 0.79	**<0.001**	0.76***	0.66, 0.88	**<0.001**
*Dry/ (0–29)*	0.26***	0.22, 0.31	**<0.001**	0.27***	0.23, 0.32	**<0.001**
*Dry/ (30–99)*	0.20***	0.18, 0.23	**<0.001**	0.25***	0.22, 0.29	**<0.001**
*Dry/ (100–199)*	0.88	0.77, 1.01	**0.087**	1.04	0.90, 1.21	0.949
*Dry/ (200–299)*	1.29***	1.11, 1.50	**<0.001**	1.37***	1.18, 1.60	**<0.001**
**Breed**						
*SIM/ GH*	1.25*	1.03, 1.52	**0.021**	1.26*	1.01, 1.57	**0.035**
*Mixed/ GH*	1.16*	1.01, 1.34	**0.035**	1.26**	1.08, 1.46	**0.001**
*Mixed/ SIM*	0.93	0.75, 1.16	0.738	1.00	0.78, 1.27	>0.999
**ECM yield**						
*(20-35)/ < 20*	0.95	0.84, 1.06	0.524	0.92	0.81, 1.04	0.230
*> 35/ < 20*	2.38***	2.10, 2.70	**<0.001**	1.76***	1.52, 2.04	**<0.001**
*> 35/ (20-35)*	2.51***	2.32, 2.72	**<0.001**	1.92***	1.75, 2.10	**<0.001**
**Housing**						
*Restrictive/ loose*	1.40**	1.10, 1.77	**0.006**	1.36*	1.04, 1.77	**0.024**
**Parity**						
*2/ 1*	0.96	0.88, 1.06	0.602	0.72***	0.65, 0.80	**<0.001**
*(3+)/ 1*	1.38***	1.27, 1.49	**<0.001**	1.00	0.92, 1.10	0.993
*(3+)/ 2*	1.43***	1.31, 1.56	**<0.001**	1.40***	1.27, 1.53	**<0.001**
**Condition**						
*Overconditioned/ normal*	0.76***	0.70, 0.83	**<0.001**	0.82***	0.74, 0.90	**<0.001**
*Underconditioned/ normal*	1.26***	1.14, 1.38	**<0.001**	1.31***	1.18, 1.45	**<0.001**
*Underconditioned/ overconditioned*	1.65***	1.46, 1.86	**<0.001**	1.60***	1.41, 1.82	**<0.001**
**Lameness**						
*Not lame/ lame*	0.81***	0.76, 0.86	**<0.001**	0.89***	0.84, 0.95	**<0.001**

N = 76,809. ORs represent the odds of ketosis risk for the first listed category (numerator) relative to the second (denominator). Significant predictors are marked: *p < 0.05, **p < 0.01, ***p < 0.001. Models account for random effects of farm.

^1^*p < 0.05; **p < 0.01; ***p < 0.001.

Abbreviations: CI = Confidence Interval, OR = Odds Ratio.

The predicted probability of KR peaked in early lactation, with 13.2% [95% CI 11.9–14.6] at DIM 0−29 and 14.0% [95% CI 12.6–15.5] at mid-early lactation (DIM 30−99). The risk decreases significantly thereafter, dropping to 3.8% [95% CI 3.41–4.17] in mid-late lactation (DIM 100−199) and reaching its lowest at 2.9% [95% CI 2.59–3.22] in late lactation (DIM 200−299). In dry cows (DIM < 0 or >299), the probability rises slightly to 3.9% [95% CI 3.52–4.36]. There is no significant difference in KR between the first two lactation stages (OR 1.07 [95% CI 0.92–1.25], p = 0.729). The odds of KR in early lactation (DIM 0−29) are 74% higher than in mid-late lactation (DIM 100−199, OR 0.26 [95% CI 0.22–0.31], p < 0.001), and 80% higher than in late lactation (DIM 200−299, OR 0.20 [95% CI 0.16–0.23], p < 0.001). In the dry period (DIM < 0 or >299), the odds are 37% higher than at DIM 200−299 (OR 1.37 [95% CI 1.18–1.60], p < 0.001) but remain significantly lower compared to early lactation (DIM 0−29, OR 0.27 [95% CI 0.23–0.32]).

German Holstein Friesian (GH) cows exhibited the lowest predicted probability of KR at 4.7% [95% CI 4.33–5.18], while Simmental (SIM) and mixed breeds had higher probabilities of 5.9% [95% CI 5.04–6.88] and 5.9% [95% CI 5.15–6.71], respectively. Compared to GH, SIM cows have 1.26 times higher odds of KR (OR 1.26 [95% CI 1.01–1.57], p = 0.035), and mixed breeds have 1.26 times higher odds (OR 1.26 [95% CI 1.08–1.46], p = 0.001).

Dairy cows with low (<20 kg) and average (20–35 kg) ECM yield, had estimated KR probability of 4.5% [95% CI 4.03–5.11] and 4.2% [95% CI 3.83–4.56], respectively. High-yielding cows (ECM yield >35 kg) show a significantly higher KR probability of 7.7% [95% CI 7.01–8.47]. The odds of KR are comparable (OR 0.92 [95% CI 0.81–1.04], p = 0.230) for cows with low or average ECM yield, while high yielding cows show odds of KR 96% higher than low-yielding cows (OR 1.96 [95% CI 1.52–2.04], p < 0.001) and 113% increased odds than average-yielding cows (OR 2.13 [95% CI 1.75–2.10], p < 0.001)

Cows in loose housing have a predicted probability of KR of 4.9% [95% CI 4.50–5.32], while those in restrictive housing have a slightly higher risk of 6.5% [95% CI 5.08–8.33]. That translates to 36% increased odds of KR (OR 1.36 [95% CI 1.04–1.77], p = 0.024) for cows in tie stalls.

The predicted probability of KR in the first parity is estimated at 5.3% [95% CI 4.86–5.85]. In contrast, cows in their second lactation (parity 2) exhibit a reduced risk, with a predicted probability of 3.9% [95% CI 3.53–4.30]. This corresponds to a 39% increase in the odds of KR for first parity cows compared to second-parity cows (OR 1.39 [95% CI 1.26–1.54], p < 0.001). Multiparous cows (parity 3 or greater) demonstrate a predicted probability of KR of 5.4% [95% CI 4.90–5.85]. That translates to 40% higher odds of KR relative to second-parity cows (OR 1.4 [95% CI 1.27–1.53], p < 0.001). However, the odds of KR in multiparous cows are statistically comparable to those observed in first parity cows (OR 1.00 [95% CI 0.92–1.10], p = 0.993), indicating no significant difference between these two groups. Notably, multivariable models account for the influence of confounding factors, which alters the associations compared to univariable models. For instance, the univariable models identified a significant difference in the probability of KR between primiparous (parity 1) and multiparous (parity 3+) cows (OR 1.37 [95% CI 1.26–1.48], p < 0.001), while the difference between first parity and second parity was not significant (OR 0.99 [95% CI 0.90–1.08], p = 0.963; Table 2).

Cows with normal body condition have a predicted probability of KR of 5.0% [95% CI 4.59–5.44]. Overconditioned cows have a lower risk of 4.1% [95% CI 3.70–4.56]. The odds of KR are 18% lower for overconditioned cows than the odds of normal-conditioned cows (OR 0.82 [95% CI 0.74–0.90], p < 0.001). Underconditioned cows exhibit the highest risk at 6.4% [95% CI 5.80–7.13]. Thus, the odds of KR are 31% higher for underconditioned cows than normal-conditioned cows (OR 1.31 [95% CI 1.18–1.45], p < 0.001) and 60% higher than overconditioned cows (OR 1.60 [95% CI 1.41–1.82], p < 0.001).

Lame cows have a predicted probability of KR of 5.3% [95% CI 4.81–5.79], while non-lame cows show a predicted KR probability of 4.7% [95% CI 4.35–5.16]. This means non-lame cows have 11% lower odds of KR compared to lame cows (OR 0.89 [95% CI 0.84–0.95], p = 0.001).

### Two-way interaction results

**Fig 3:**
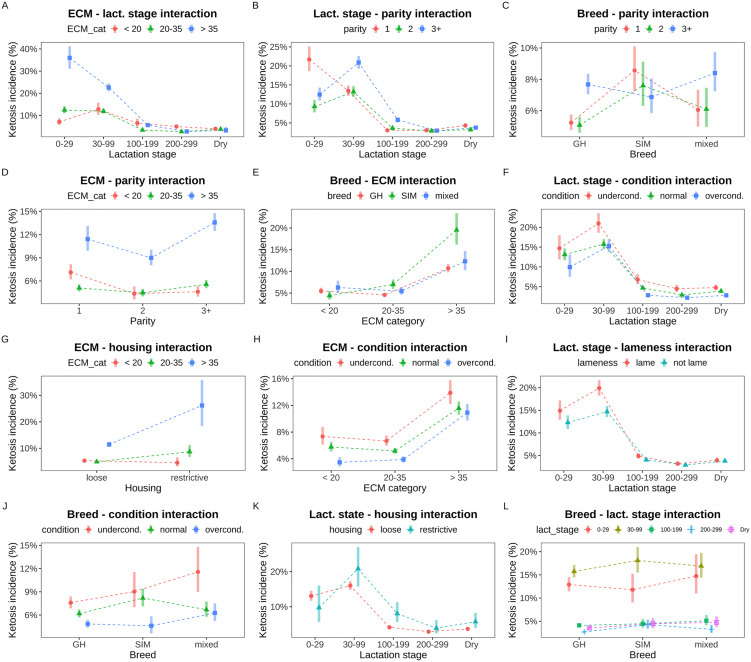
Significant two-way interactions for ketosis risk factors. (N = 76,809). Twelve significant two-way interactions were identified and are shown in order of statistical relevance (p-value) from most to least significant (Panels A – L), based on Type II Wald Chi-Square analysis of deviance. Each panel illustrates the combined influence of two variables on the predicted probability of ketosis risk (%), with points representing mean predicted probabilities and error bars indicating 95% confidence intervals. Probabilities are adjusted for all other fixed and random effects in the multivariable model. Colors denote categories of the interacting variables; abbreviations: “ECM” = Energy corrected milk yield, “Lact. stage” = Lactation stage, “undercond.” = underconditioned, “overcond.” = overconditioned.

Twelve two-way interactions were identified as having a significant relevance for KR. These interactions are illustrated in Fig 3 and are described below in descending order of their relative association with the risk of ketosis.

#### Interaction between ECM yield and lactation stage.

The interaction between ECM yield and lactation stage (Fig 3, panel A) turned out to be the most influential on KR (χ² = 201, p < 0.001). In the early lactation (DIM 0–29), high-yielding cows (ECM yield > 35) exhibit the highest predicted probability of KR within this interaction at 36.0% [95% CI 31.1–41.2], average-yielding cows (ECM yield 20–35) have a lower probability of 12.5% [95% CI 11.0–14.2] and low-yielding cows (ECM yield < 20) show the lowest predicted probability in early lactation of 7.1% [95% CI 5.8–8.8]. Therefore, high-yielding cows have 294% increased odds compared to average-yielding cows (OR 3.94 [95% CI 2.96–5.25], p < 0.001) and 631% increased odds of KR compared to low-yielding cows (OR 7.31 [95% CI 5.11–10.46], p < 0.001). Average-yielding cows have 85% higher odds of KR than low-yielding cows (OR = 1.85 [95% CI 1.38–2.49], p < 0.001).

The difference between low and average yielding cows vanishes in mid-early lactation (DIM 30–99), while high-yielding cows remain significantly higher in their odds of KR. When looking at the transition from early to mid-early lactation, the probability for high-yielding cows decreases significantly to 22.6% [95% CI 20.9–24.5], average-yielding cows exhibit a probability of KR at 11.9% [95% CI 10.9–13.1], and low-yielding cows show a probability of KR of 12.8% [95% CI 10.3–15.8]. The predicted probability for high-yielding cows in mid-early lactation indicates a 48% reduction in odds from DIM 0–29 (OR = 0.52 [95% CI 0.39–0.70], p < 0.001). The odds of KR in average-yielding cows remain stable in the mid-early lactation period (OR 0.95 [95% CI 0.78–1.16], p = 0.956), while low-yielding cows experience a 91% increase in odds of KR (OR 1.91 [95% CI 1.23–2.96], p < 0.001).

Beyond DIM 99, the risk of KR decreases significantly across all ECM yield categories. Since KR is primarily relevant in earlier lactation stages, comprehensive details on probabilities, ORs, CIs, and p-values for DIM 99+ and the dry period for all interactions with the lactation stage are provided in S2 File.

#### Interaction between parity and lactation stage.

The interaction between parity and lactation stage (Fig 3, panel B, χ² = 162, p < 0.001) shows primiparous cows in early lactation (DIM 0–29) exhibiting the highest predicted probability of KR at 21.7% [95% CI 18.6–25.1]. In contrast, second-parity cows had a predicted probability of KR of 9.3% [95% CI 7.8–11.1], while multiparous cows (parity 3+) showed a 12.5% risk [95% CI 10.8–14.3]. The odds of KR for primiparous cows in early lactation were 171% increased (OR 2.71 [95% CI 2.0–3.7], p < 0.001) compared to second-parity cows and 95% higher (OR 1.95 [95% CI 1.45–2.55], p < 0.001) than for multiparous cows.

In mid-early lactation (DIM 30–99), the pattern shifted, with multiparous cows demonstrating the highest probability of KR at 21.0% [95% CI 19.3–22.6]. Primiparous and second-parity cows exhibited similar risks, with predicted probabilities of 13.5% and 13.2%, respectively. Their odds of KR are comparable (OR = 1.0, p = 0.94). The odds of KR in multiparous cows were 70% higher compared to primiparous cows (OR 1.7 [95% CI 1.49–1.94], p < 0.001) and 74% increased compared to second-parity cows (OR 1.74 [95% CI 1.51–2.01], p < 0.001).

From early to mid-early lactation, the probability of KR in primiparous cows decreased significantly to 13.5% [95% CI 12.2–14.9] and further declined to 3.1% [95% CI 2.6–3.5] in mid-late lactation (DIM 100–199). Thus, the odds of KR are 44% lower for primiparous cows in mid-early lactation compared to early lactation (OR 0.56 [95% CI 0.43–0.74], p < 0.001). Conversely, second-parity cows experienced a 48% increase in KR odds during the transition from early to mid-early lactation (OR 1.48 [95% CI 1.12–1.97], p = 0.001). Multiparous cows showed an even higher increase of 86% in KR odds from early to mid-early lactation (OR 1.86 [95% CI 1.5–2.3], p < 0.001). Despite this elevated risk, the probability of KR in secundiparous and multiparous cows dropped significantly after DIM 99 and remained low throughout the lactation.

#### Interaction between breed and parity.

The interaction between breed and parity (Fig 3, panel C, χ² = 48, p < 0.001), reveals distinct patterns in the risk of ketosis. GH in the first parity and second-parity GH cows exhibit similarly low KR probabilities (5.2% [CI 4.8–5.8] and 5.1% [95% 95% CI 4.6–5.6], respectively). This translates to almost identical odds of KR (OR 1.03 [95% CI 0.93–1.15], p = 0.477). In contrast, multiparous GH cows (parity 3+) show a higher probability of 7.7% [95% CI 7.1–8.4]. Their odds are 50% higher than those in parity 1 (OR 1.50 [95% CI 1.37–1.65], p < 0.001) and 55% higher than those in parity 2 (OR 1.55 [95% CI 1.41–1.72], p < 0.001).

Mixed-breed cows follow a similar trend, with probabilities of 6.1% [95% CI 5.0–7.3] in parity 1, 6.1% [95% CI 5.0–7.5] in parity 2 and increasing to 8.4% [95% CI 7.2–9.7] in parity 3 + .

SIM cows, however, display a decreasing trend: 8.6% [95% CI 7.3–10.1] in parity 1, 7.6% [95% CI 6.3–9.1] in parity 2, and 6.9% [95% CI 5.9–8.1] in parity 3 + . This shows odds 27% higher for primiparous compared to multiparous cows (OR 1.27 [95% CI 1.02–1.59], p = 0.031). Odds differences between parity 1 and 2 (OR 1.14 [95% CI 0.88–1.47], p = 0.286) and parity 2 and 3+ (OR 1.12 [95% CI 0.87–1.42], p = 0.286) are not significant, however.

Across breeds, SIM cows in the first parity have 70% higher odds than respective GH cows (OR 1.70 [95% CI 1.33–2.16], p < 0.001) and 45% higher odds than mixed-breed primiparous cows (OR 1.45 [95% CI 1.05–2.02], p = 0.009). SIM second-parity cows have 54% higher odds than GH counterparts (OR 1.54 [95% CI 1.18–2.00], p < 0.001).

#### Interaction between ECM yield and parity.

The ECM yield and parity interaction (Fig 3, panel D, χ² = 44, p < 0.001) highlights high-yielding cows (ECM yield > 35) with the highest risk across parities: 11.4% [95% CI 9.9–13.1] in parity 1, decreasing to 9% [95% CI 8.0–10.0] in parity 2, and rising to a probability of 13.6% [95% CI 12.5–14.8] in multiparous cows. High-yielding cows decrease their odds of KR by 23% when comparing primiparous with second-parity cows (OR 0.77 [95% CI 0.63–0.93], p = 0.004), while the odds rise by 22% for multiparous cows compared to primiparous cows (OR 1.22 [95% CI 1.02–1.45], p = 0.023).

Average-yielding cows (ECM yield 20–35) have the lowest risk of ketosis at 5.1% [95% CI 4.6–5.6] in parity 1, with stable predicted probabilities in parity 2 (prob. = 4.5% [95% CI 4.0–5.0]) and in multiparous cows (prob. = 5.6% [95% CI 5.1–6.1]). This translates to comparable odds of KR for primiparous and second-parity cows (OR 0.87 [95% CI 0.77–0.99], p = 0.035) as well as cows in parity 1 and parity 3+ (OR 1.1 [95% CI 0.99–1.23], p = 0.105).

Low-yielding cows (ECM yield < 20) show a predicted probability of KR of 7.1% [95% CI 6.2–8.2] as primiparous cows, dropping the predicted probability to 4.4% [95% CI 3.6–5.3] in parity 2, and remaining low in multiparous cows (4.6% [95% CI 4.0–5.3]). Thus, low-yielding cows in the first parity decrease the odds of KR by 41% in parity 2 (OR 0.59 [95% CI 0.45–0.78], p < 0.001) while the odds remain stable comparing parity 2 and parity 3+ (OR 1.06 [95% CI 0.81–1.39], p = 0.869).

Thus, multiparous high-yielding cows have 225% higher odds than low-yielding counterparts (OR 3.25 [95% CI 2.71–3.91], p < 0.001). The odds of KR for low-yielding primiparous cows are 43% higher than average-yielding counterparts (OR 1.43 [95% CI 1.2–1.71], p < 0.001) but 40% lower than high-yielding cows in the first parity (OR 0.6 [95% CI 0.47–0.75], p < 0.001).

#### Interaction between ECM yield and breed.

The interaction between breed and ECM yield (Fig 3, panel E, χ² = 39, p < 0.001) shows that at low ECM yields (< 20 kg), GH and SIM cows exhibit predicted probabilities of 5.5% [95% CI 4.8–6.2] and 4.4% [95% CI 3.5–5.5], respectively. Therefore, their odds of KR are similar (OR 0.79 [95% CI 0.58–1.09], p = 0.205).

In contrast, at average ECM yields (20−35 kg), SIM cows have a higher probability of 7.0% [95% CI 6.0–8.1] versus 4.6% [95% CI 4.2–5.0] for GH. Their odds of KR are 56% higher compared to GH cows (OR 1.56 [95% CI 1.25–1.95], p < 0.001).

This disparity widens at high ECM yields (> 35 kg), where SIM cows exhibit a predicted probability of 19.5% [95% CI 16.2–23.4] and 10.7% [95% CI 9.8–11.7] for GH cows. This corresponds to 102% higher odds for SIM than GH cows (OR 2.02 [95% CI 1.51–2.72], p < 0.001).

For GH cows, the lowest KR occurs at average ECM yields (prob. = 4.6% [95% CI 4.2–5.0]), with odds 17% lower than at low ECM yields (OR 0.83 [95% CI 0.72–0.95], p = 0.004) and 59% lower than at high ECM yields (OR 0.41 [95% CI 0.31–0.54], p < 0.001). Conversely, SIM cows exhibit their lowest risk at low ECM yields (prob. = 4.4% [95% CI 3.5–5.5]), which increases to 7.0% [95% CI 6.0–8.1] at average ECM yields (OR 1.63 [95% CI 1.25–2.14], p < 0.001) and rises sharply to 19.5% [95% CI 16.2–23.4] at high ECM yields (OR 5.29 [95% CI 3.76–7.43], p < 0.001). These patterns suggest that GH cows experience a minimal risk of ketosis at moderate milk yields, whereas SIM cows face an escalating risk as production increases.

Mixed-breed cows follow a risk profile similar to GH cows, with their lowest risk at average ECM yields (5.4% [95% CI 4.6–6.4]; additional details are provided in S2 File).

#### Interaction between lactation stage and condition.

In the interaction between body condition and lactation stage (Fig 3, panel F, χ² = 32, p < 0.001) the highest KR is observed during the first 99 days of lactation. In early lactation (DIM 0–29), underconditioned cows exhibit the highest probability of KR at 14.7% [95% CI 11.9–18.0], while overconditioned cows show a probability of 10.0% [95% CI 7.5–13.1], and optimally conditioned cows have a probability of 13.1% [95% CI 11.7–14.7]. The odds increase, therefore, by 56% for underconditioned cows compared to overconditioned cows (OR 1.56 [95% CI 0.99–2.45], p = 0.057) but are not significantly higher for optimally conditioned cows (OR 1.14 [95% CI 0.84–1.55], p = 0.565). Optimally conditioned cows have 37% higher odds than overconditioned cows (OR 1.37 [95% CI 0.93–2.01], p = 0.083).

In mid-early lactation (DIM 30–99), underconditioned cows reach a peak probability of 21.0% [95% CI 18.6–23.5]. Overconditioned cows have a lower predicted probability of 15.3% [95% CI 13.7–17.0], while optimally conditioned cows exhibit a comparable level at 15.7% [95% CI 14.5–17.0]. This translates to 47% higher odds for underconditioned than for overconditioned cows (OR 1.47 [95% CI 1.21–1.79], p < 0.001) and 42% higher odds than for optimally conditioned cows (OR 1.42 [95% CI 1.20–1.69], p < 0.001). Optimally conditioned cows show a 24% increase in odds from DIM 0–29 to DIM 30–99 (OR 1.24 [95% CI 1.04–1.47], p = 0.006) and have similar odds to overconditioned cows (OR 1.04 [95% CI 0.90–1.19], p = 0.549).

#### Interaction between ECM yield and housing.

In the interaction between ECM yield and housing type (Fig 3, panel G, χ² = 18, p < 0.001), low-yielding (ECM yield < 20kg) and average-yielding cows (ECM yield 20−35 kg) in free-stall housing exhibit similar probabilities of KR, at 5.4% [95% CI 4.8–6.0] and 4.9% [95% CI 4.5–5.3], respectively. High-yielding cows exhibit higher predicted probabilities of KR at 11.5% [95% CI 10.6–12.4]. The odds of KR are comparable for low- and average-yielding cows (OR 1.10 [95% CI 0.97–1.24], p = 0.067). High-yielding cows (ECM yield > 35 kg) in free-stall housing have 151% higher odds than average-yielding cows (OR 2.51 [95% CI 2.32–2.72], p < 0.001).

In restrictive housing, high-yielding cows (ECM yield > 35 kg) exhibit the highest risk, with a probability of 26.2% [95% CI 18.5–35.8]. Average-yielding cows in restrictive housing show a probability of 8.7% [95% CI 6.8–11.2]. Low-yielding cows in restrictive housing have a stable probability of 4.6% [95% CI 3.1–6.6], with no significant difference in odds compared to loose housing (OR 0.85 [95% CI 0.57–1.27], p = 0.417), indicating a minimal role of housing type for this subpopulation. High-yielding cows, however, increase their odds by 174% when kept restrictively (OR 2.74 [95% CI 1.74–4.33], p < 0.001); average-yielding cows also increase their odds by 85% when kept restrictively (OR 1.85 [95% CI 1.39–2.47], p < 0.001).

The greatest divergence occurs between average- and high-yielding cows in restrictive housing, with high-yielding cows having 272% higher odds (OR 3.72 [95% CI 2.17–6.36], p < 0.001).

#### Interaction between ECM yield and body condition.

The interaction between ECM yield and body condition (Fig 3, panel H, χ² = 19, p < 0.001) consistently shows that underconditioned cows have the highest risk across all ECM yield categories. Low-yielding underconditioned cows (ECM yield < 20 kg) have a KR probability of 7.4% [95% CI 6.1–8.8], at average yield (ECM yield 20–35 kg) the probability is 6.7% [95% CI 6.0–7.5] and at high yield (ECM yield > 35 kg) it is 13.9% [95% CI 12.2–15.8]. Thus, the odds are not significantly reduced for underconditioned average-yielding cows compared to low-yielding cows (OR 0.90 [95% CI 0.71–1.15], p = 0.577), but 103% higher for high-yielding cows compared to low-yielding cows (OR 2.03 [95% CI 1.56–2.66], p < 0.001).

For overconditioned cows, low-yielding cows show a probability of 3.5% [95% CI 2.8–4.2], average-yielding cows stand at 3.9% [95% CI 3.5–4.4], and high-yielding overconditioned cows exhibit a predicted probability of KR of 10.9% [95% CI 9.7–12.2]. This indicates that underconditioned cows always display higher odds of KR with 120% increase at low yield (OR 2.20 [95% CI 1.61–3.02], p < 0.001), 77% increase of the odds for average ECM yield (OR 1.77 [CI 1.50–2.09], p < 0.001) and lastly 32% increased odds at high yield (OR 1.32 [95% CI 1.08–1.60], p = 0.003).

Optimally conditioned cows exhibit intermediate risks, with probabilities of 5.8% [95% CI 5.1–6.5] for low-yielding and 5.2% [95% CI 4.8–5.6] for average-yielding cows, and for high-yielding cows 11.6% [95% CI 10.6–12.6]. The odds are therefore comparable for low- and average-yielding cows when optimally conditioned (OR 1.12 [95% CI 0.97–1.31], p = 0.063). Optimally conditioned high-yielding cows have 140% higher odds compared to average-yielding cows (OR 2.40 [95%CI 2.18–2.65], p < 0.001).

#### Interaction between lactation stage and lameness.

Lame cows consistently show a higher risk of ketosis than non-lame cows in the interaction between lactation stage and lameness (Fig 3, panel I, χ² = 17, p = 0.002). In early lactation (DIM 0−29), lame cows have a probability of 14.9% [95% CI 12.9–17.2] versus 12.3% [95% CI 10.9–13.9] for non-lame cows. Their odds of KR are therefore 25% higher than non-lame cows (OR 1.25 [95% CI 1.04–1.51], p = 0.02).

During mid-early lactation (DIM 30−99), lame cows exhibit a higher probability than non-lame cows with 19.9% [95% CI 18.3–21.7] and 14.7% [95% CI 13.5–15.9], respectively. Thus, their odds are 44% higher than non-lame cows (OR 1.44 [95% CI 1.31–1.59], p < 0.001). In this lactation stage, non-lame cows’ odds increase by 23% compared to early lactation (OR 1.23 [95% CI 1.02–1.48], p = 0.02), while lame cows’ odds rise more significantly by 42% (OR 1.42 [95% CI 1.12–1.78], p < 0.001).

#### Interaction between breed and body condition.

The interaction between breed and body condition (Fig 3, panel J, χ² = 16, p = 0.003) shows overconditioned GH and SIM cows exhibiting similar low probabilities of KR at 4.9% [95% CI 4.4–5.4] and 4.6% [95% CI 3.6–5.8], respectively. Their odds of KR are comparable (OR 1.06 [95% CI 0.76–1.47], p = 0.697). Mixed-breed cows, however, exhibit a higher probability of KR at 6.3% [95% CI 5.2–7.5] when overconditioned.

Optimally conditioned GH cows have a probability of 6.2% [95% CI 5.7–6.7], while respective SIM cows have a probability of 8.2% [95% CI 7.1–9.4]. Mixed breeds maintain a comparable level of KR when optimally conditioned at 6.7% [95% CI 5.8–7.8]. The odds are 29% increased for GH compared to overconditioned counterparts (OR 1.29 [95% CI 1.16–1.44], p < 0.001) and 85% increased for SIM when optimally conditioned compared to overconditioned (OR 1.85 [95% CI 1.39–2.46], p < 0.001). Thus, optimally conditioned SIM cows have 36% higher odds than their GH counterparts (OR 1.36 [CI 1.11–1.66], p = 0.001).

Underconditioned cows exhibit the highest risk of ketosis: GH cows have a probability of 7.6% [95% CI 6.9–8.4], SIM cows 9.0% [95% CI 7.0–11.6], and mixed-breed cows show the highest probability at 11.6% [95% CI 9.0–14.8]. The odds of KR are 59% higher for mixed breed cows than underconditioned GH cows (OR 1.59 [95% CI 1.13–2.24], p = 0.004).

The odds of KR are 61% higher for underconditioned GH cows compared to overconditioned counterparts (OR 1.61 [95% CI 1.41–1.84], p < 0.001), and 105% higher for the corresponding SIM comparison (OR 2.05 [95% CI 1.38–3.06], p < 0.001).

#### Interaction between lactation stage and housing.

Fig 3, panel K shows the two-way interaction of lactation stage and housing. In early lactation (DIM 0–29), cows in loose housing have a higher KR probability of 13.1% [95% CI 11.7–14.5] compared to those in restrictive housing at 9.7% [95% CI 5.7–16.0], though the difference is not significant (OR 1.39 [95% CI 0.78–2.49], p = 0.264).

In mid-early lactation (DIM 30−99), however, cows in restrictive housing with a probability of KR of 20.8% [95% CI 15.8–27.0] have a notably higher predicted probability of KR than those in free-stall housing with 16.0% [95% CI 14.9–17.3]. The odds are 38% higher for restrictively housed cows compared to cows in free-stall housing (OR 1.38 [95% CI 0.97–1.96], p = 0.071). Cows in tie-stall housing exhibit a 144% increase in their odds of KR compared to early lactation (DIM 0−29, OR 2.44 [95% CI 1.04–5.71], p = 0.034), while cows in loose housing only experience a 27% increase in their odds (OR 1.27 [95% CI 1.1–1.47], p < 0.001).

#### Interaction between lactation stage and breed.

The least significant interaction (χ² = 15, p = 0.018) is between breed and lactation stage (Fig 3, panel L). In early lactation (DIM 0–29), GH cows have a KR probability of 12.9% [95% CI 11.5–14.5], comparable to SIM with 11.8% [95% CI 9.1–15.2]. Their odds of KR are consequently similar (OR 1.11 [95% CI 0.75–1.65], p = 0.528). Mixed breeds exhibit a predicted probability of KR of 14.7% [95% CI 11.0–19.4] in early lactation.

All breeds then show rising tendencies in KR probability in mid-early lactation (DIM 30–99). GH shows a predicted probability of KR at 15.7% [95% CI 14.5–17.1]. This translates to a 26% increase in the odds of KR (OR 1.26 [95% CI 1.07–1.48], p < 0.001) compared to early lactation. SIM cows have a significantly higher predicted probability of KR, rising to 18.1% [95% CI 1.09–2.50]. This results in a 65% increase in their odds of KR (OR 1.65 [95% CI 1.09–2.50], p = 0.008) compared to early lactation. The least significant rise in KR probability to 16.9% [95% CI 14.4–19.8] is shown by mixed-breed cows. Their odds increase by only 18% (OR 1.18 [95% CI 0.72–1.94], p = 0.898), which is not significant, with notably overlapping 95% CIs in early and mid-lactation.

### Multivariable scenarios

To assess practical implications, multivariable predicted probabilities of KR were computed for hypothetical cows across various scenarios, including best- and worst-case scenarios (Table 3). Scenarios apart from the best and worst cases were averaged across housing and lactation stages.

**Table 3:** Predicted ketosis risk across hypothetical cow scenarios.

Scenario	Parameters	Breed	Predicted ketosis Risk [95% CI] (%)
A: best case	ECM yield = 20–35, parity = 2, condition = overconditioned,	GH	1.5 [1.3-1.7]
	lameness = not lame	SIM	1.8 [1.5-2.2]
	housing = loose, DIM = 200-299	mixed	1.8 [1.5-2.2]
B: worst case	ECM yield > 35, parity = 1 condition = underconditioned	GH	34.4 [28.1-41.4]
	lameness = lame	SIM	39.8 [33.1-47]
	housing = restrictive, DIM = 30-99	mixed	39.8 [32.6-47.4]
C: healthy older cow, high yield	ECM yield > 35, parity = 3+	GH	9.4 [8.4-10.4]
	condition = normal	SIM	11.5 [9.8-13.5]
	lameness = not lame	mixed	11.5 [10-13.2]
D: lame, undercond. older cow, high yield	ECM yield > 35, parity = 3+	GH	13.3 [11.9-14.8]
	condition = underconditioned	SIM	16.2 [13.7-18.9]
	lameness = lame	mixed	16.1 [14-18.5]
E: lame, overcond. heifer, high yield	ECM yield > 35, parity = 1	GH	9 [7.9-10.3]
	condition = overconditioned	SIM	11.1 [9.2-13.4]
	lameness = lame	mixed	11.1 [9.4-13]

Hypothetical risk scenarios were generated from the multivariable model by specifying combinations of predictor variables: breed (German Holstein [GH], Simmental [SIM], mixed), body condition (underconditioned, normal, overconditioned), milk yield categories (ECM yield: < 20, 20–35, > 35 kg), parities (1, 2, ≥ 3), lameness status, housing type (loose, restrictive), and lactation stage (days in milk [DIM]). Scenarios A (best-case) and B (worst-case) represent extreme risk profiles, while scenarios C-E reflect intermediate conditions averaged across housing and lactation stages unless explicitly stratified. Abbreviations: “undercond.” = underconditioned, “overcond.” = overconditioned.

The best-case scenario (Table 3, A) shows the lowest KR for GH and SIM cows at 1.4% [95% CI 1.2% − 1.7%] and 1.8% [95% CI 1.5% − 2.2%], respectively, occurring in non-lame, overconditioned second-parity cows in free-stall housing in late lactation (DIM 200–299) and average milk yield (ECM yield 20–35). The worst-case scenario (Table 3, B) indicates the highest risk of ketosis at 34.2% [95% CI 27.8% − 41.2%] for GH and 39.8% [95% CI 33.0% − 47.0%] for SIM, seen in lame, underconditioned high-yielding (ECM yield > 35) first-parity cows in mid-early lactation (DIM 30–99) with restrictive housing.

Scenario C (Table 3, C) represents a non-lame, typically conditioned older cow (parity ≥ 3) with high milk yield (ECM yield > 35 kg). This scenario describes an intermediate-risk profile with predicted KR probabilities of 9.4% [95% CI 8.4% − 10.4%] for GH and 11.5% [95% CI 9.8% − 13.5%] for SIM. Mixed-breed cows show a similar risk level at 11.5% [95% CI 10.0% − 13.2%].

Scenario D (Table 3, D) involves lame, underconditioned older cows (parity ≥ 3) with high milk yield (ECM yield > 35 kg). This scenario reflects a higher intermediate risk, with predicted KR probabilities of 13.3% [95% CI 11.9% − 14.8%] for GH and 16.2% [95% CI 13.7% − 18.9%] for SIM, and 16.1% [95% CI 14.0% − 18.5%] for mixed-breed cows.

Scenario E (Table 3, E) corresponds to a lame, overconditioned primiparous cow (parity = 1) with high milk yield (ECM yield > 35 kg). This profile presents another intermediate-risk scenario with predicted probabilities of 9.0% [95% CI 7.9% − 10.3%] for GH, 11.1% [95% CI 9.2% − 13.4%] for SIM, and 11.1% [95% CI 9.4% − 13.0%] for mixed-breed cows.

## Discussion

This study aimed to identify key animal-level predictors for KR in German dairy cows and to assess how these factors interact across breeds, parities, and management systems using the described alternative method to detect KR via milk-record data without the need to test for β-hydroxybutyrate (BHB) blood levels. Our analysis revealed that KR is strongly associated with lactation stage, ECM yield, parity, and body condition, with additional contributions from breed, lameness, and housing conditions. Importantly, these factors do not act in isolation; instead, they interact in complex, often non-linear ways. We identified particularly vulnerable groups, including high-yielding cows in early lactation, underconditioned cows in mid-early lactation, and SIM cows, especially primiparous, at high production levels. Conversely, favorable conditions, for example, optimal body condition and absence of lameness, mitigate risk even in high-yielding or multiparous cows. Overall, our findings underscore that a single factor does not determine KR susceptibility; rather, it is significantly shaped by dynamic interactions among physiology, health, welfare, and management.

### Data handling

The results of univariable mixed-effects logistic regressions are included in our results section solely for comparison with prior or future studies that may employ univariable analyses. Given the multifactorial nature of KR, the multivariable mixed-effects logistic regression better reflects the complexity of risk factors in this dataset [13,44]. Thus, we focused on describing multivariable and two-way interaction results and are also using these as the foundation of our discussion.

The Random Forest analysis was prioritized for ranking risk factors due to its ability to relax assumptions of classic statistical models, e.g., to capture non-linear relationships among predictors, which are likely present in the complex etiology of KR, such as the interplay between lactation stage, ECM yield, and body condition [45]. While the multivariable mixed-effects logistic regression provides interpretable odds ratios and accounts for hierarchical data, its assumption of linearity may overlook nuanced patterns that Random Forest can detect, making the latter more suitable for identifying the relative importance of risk factors in this multifactorial context [13].

### Days in milk

Our data demonstrate that the highest risk period for KR extends well into mid-early lactation, indicating that monitoring should not be limited to the traditional early lactation period (DIM 0–21) and suggests an extended window of metabolic susceptibility lasting up to 14 weeks after calving.

The lack of decline in KR probability from DIM 0–29 to DIM 30–99 implies that many cows fail to achieve appropriate metabolic adaptation even after the immediate post-calving phase. This prolonged state likely reflects ongoing NEB due to insufficient feed intake relative to escalating milk production demands [10,46]. Continued lipolysis and incomplete oxidation of non-esterified fatty acids (NEFAs) sustain elevated BHB levels [47], contributing to a persistent risk of ketosis. This manifests an extended risk period, where physiological recovery lags behind production increases, particularly in high-yielding cows [48,49]. Thus, the absence of a drop in predicted KR until DIM 99 in our results emphasizes the importance of nutritional and management strategies that support transition success over several weeks.

Importantly, to our knowledge, no studies have systematically assessed KR beyond 60 DIM. Our observations support the persistence of metabolic vulnerability. The biological plausibility of prolonged energy deficit in high-producing cows suggests that what we observe is a continuum of poor metabolic adaptation rather than isolated disease events, or possibly a combination of both, which aligns with Zhang and Ametaj’s [10] proposed categorization, describing Type I and Type II ketosis as arising from distinct physiological mechanisms in the cows’ metabolism.

### Breed

GH cows exhibit the overall lowest probability of KR at 4.7%, while SIM and mixed-breed cows have higher probabilities of 5.9% each. It seems counterintuitive that the odds of KR are lower for breeds with generally higher milk production, such as GH [50], as our results show a significantly higher risk of KR for high ECM yield (>35 kg). This contradicts the assumption that higher milk yield primarily increases ketosis susceptibility, as well as the established idea that SIM generally express a lower susceptibility to ketosis compared to Holsteins [51]. Instead, breed-specific genetic background, production level, metabolic efficiency, and management intensity may play more important roles than previously recognized.

The reduced KR in GH cows, despite their higher genetic potential for milk yield, may reflect superior metabolic adaptation, potentially involving more efficient energy metabolism or hormonal regulation, although this warrants further study. Alternatively, GH cows might benefit from closer monitoring or more intensive management. Farms with GH herds often operate on larger scales, which frequently translates to more intensive supervision and protocols, due to their economic importance. This organizational focus is likely to facilitate earlier detection and intervention for metabolic imbalances.

In contrast, SIM, usually dual-purpose cattle farmed for both milk and meat, may be managed less intensively, with potentially less emphasis on metabolic disease prevention and transition cow monitoring. This could increase vulnerability to metabolic stress. Moreover, their energy partitioning strategies may differ, potentially making them less efficient at mobilizing and utilizing body reserves during transition and early lactation phases. Zablotski et al. (2022) found that SIM cows show different BCS trajectories over their lifetime compared to those of GH, with greater fluctuations in body reserves (especially for overconditioned SIM), indicating less metabolic stability during energy-demanding periods [36]. Springer et al. (2021) further demonstrated that herd breed type (high-performance dairy vs. dual-purpose) is significantly associated with differences in the intensity of feeding, housing, and health management [52]. These findings support the argument that the lower KR for GH cows reflects a combination of superior management practices and systematic use of monitoring tools.

Thus, our data suggest that modern GH lines may have been (indirectly) genetically selected not only for high milk yield but also metabolic resilience. This contrasts with previous studies reporting that SIM cows generally exhibit lower postpartum NEFA concentrations, smaller changes in body fat and BCS during NEB [53], and more efficient restoration of BCS after peak lactation, as well as more effective liver function during NEB [54]. Our results highlight the necessity to re-evaluate assumptions regarding breed-specific KR and emphasizes the need to establish a general, continuous health reporting system with high participation to obtain a sufficient phenotypic and genotypic data for direct health traits, as previously suggested [55].

Based on these findings, we recommend reassessing breed-based risk assumptions that are currently established and to implement uniform KR testing protocols regardless of breed to avoid under-detection. When designing disease prevention programs and management protocols, breed composition should be used as an essential factor, particularly on multi-breed farms. Farmers should critically evaluate the concept of multi-breed herds in general, as managing a single breed may allow for more tailored and effective accommodation of that breed’s specific management needs. This point will benefit from further research.

### Energy-corrected milk (ECM) yield

In our analysis, high-yielding cows had 1.96 times the odds of KR compared to low-yielding cows, highlighting the significant association of production intensity with metabolic health. This result aligns with the established understanding that high milk yield is correlated with increased NEB postpartum and metabolic stress [56]. A retrospective cohort study also found that for GH cattle, each additional 1,000 kg of milk from previous lactations significantly increased the odds of hyperketonemia in the following early lactation [18]. These findings suggest that genetic selection for high-yield, particularly in high-producing breeds, may predispose cows to metabolic imbalance. Recent evidence suggests moderate heritability of KR [57], challenging the notion that KR is primarily management-driven.

Approximately one-third of dairy cows suffer from postpartum diseases like ketosis linked to NEB [58], contesting the view that high yield is a reasonable primary breeding goal. Economic impact assessments [59,21] underscore the urgency to investigate the balance between health costs and the financial benefits of increased milk production.

From a management perspective, our results show that keeping ECM yield below 35 kg or enhancing feed efficiency with energy supplements not only during the transition period, but also breed and age specific, appears advisable. These recommendations are consistent with established BCS optimization strategies [36]. Propylene glycol treatments have shown effectiveness in mitigating ketosis, although the long-term impacts on herd health are still under review [49,60]. Overall, further investigation into the interaction between genetics and management practices is needed to support sustainable productivity while maintaining animal welfare.

### Housing

Cows housed in tie-stalls exhibit significantly higher odds of KR (OR 1.36) compared to their counterparts in free-stall housing. A previous study, based on the same research material [22], has already linked tie-stall housing to lameness in dairy cows [61]. Thus, the role of tie-stall vs. free-stall housing in preventing metabolic disorders, such as ketosis, also deserves a detailed examination, as our results suggest that housing should be considered a standalone predictor for KR.

This aligns with findings from Norwegian dairy herds, where tie-stall systems had a significantly higher probability of ketosis than free-stall systems, as well as a general association of free-stall systems with lower risk of ketosis, reduced mastitis, and fewer teat injuries [62,63]. In addition, the housing-welfare literature further supports the benefits of loose housing, also linking it to a lower risk of ketosis and improved fertility relative to restrictive housing [64].

The elevated risk of ketosis in tie-stall cows likely stems from interrelated welfare and behavioral deficiencies. Free-stall systems promote natural feeding patterns, more frequent, smaller meals, that help maintain rumen stability, reduce the risk of subacute acidosis, and ensure a smoother energy supply [63,65,66]. Furthermore, tie-stall housing is associated with a higher risk of metabolic and health disorders compared to loose/free-stall systems, and improved welfare outcomes have been reported in free-stall housed cows, particularly regarding exercise opportunities [64,67].

Housing is a significant risk factor for metabolic diseases and should be integrated into prevention protocols. Simply adjusting feeding rations may not be sufficient if the environment promotes stress, irregular feed intake, and limited movement. Although tie-stall systems allow for easier monitoring due to cow immobilization, the risk of ketosis remains elevated, underscoring that surveillance alone cannot compensate for suboptimal housing design. These findings bridge an important gap: while most German studies focus on nutrition or genetics, few assessed housing, especially free- versus tie-stall systems, as a direct contributor to metabolic health in dairy cows.

It is important to note that the multivariable results adjust for all other predictors, thereby the reported estimates represent the independent association of each factor while controlling for confounding from other variables in the model. In practice, housing influences are more multifaceted. A well-managed tie-stall can outperform a poorly designed free-stall, but such subtleties are captured only through interactions between predictors, which are explored in a later section.

Based on the multivariable results alone, we recommend prioritizing free-stall housing, particularly during the transition and early lactation periods. Where tie-stall systems remain in use, they should be carefully evaluated or gradually replaced due to their association with restricted movement and higher metabolic risk. At a minimum, housing adaptions should provide sufficient space, comfortable stalls, opportunities for exercise, and feeding arrangements that reflect natural feeding behavior.

### Parity

Our results show that both primiparous and multiparous cows exhibit significantly higher risk of ketosis compared to second-parity cows (~5.3–5.4% vs. 3.9%). This challenges the traditional assumption that KR increases linearly with parity [6] and instead reveals a U-shaped pattern, highlighting the second parity as a period of relative metabolic resilience.

This metabolic resilience in second parity may reflect an optimal combination of physiological maturity and production capacity. First-parity cows must partition energy between growth and milk production, increasing their NEB [46]. Moreover, primiparous cows are often socially subordinate to multiparous cows, potentially limiting their access to feed resources and heightening stress associated with social interactions in free-stall housing systems. In contrast, second-parity cows achieve higher milk yields without the same degree of metabolic strain seen in older cows. Additionally, cows in their second parity may benefit from improved management focus or better rumen development compared to primiparous cows. An alternative explanation is that some primiparous cows with low yield, possibly due to undetected, prolonged SCK, or other metabolic challenges, including cases of clinical ketosis, may be culled or fail to rebreed before their second parity. As a result, the population entering second parity may represent a metabolically more resilient subset, contributing to the observed drop in KR, which then rises again in multiparous cows as milk production increases with age. This hypothesis is supported by studies showing that metabolic disease (in early lactation) is associated with increased culling and reduced reproductive success [13,68], potentially removing higher-risk animals from later parity groups.

In contrast, multiparous cows exhibit greater metabolic strain [69], likely reflecting the cumulative physiological demands of repeated lactations. These demands include reduced insulin sensitivity, higher NEFA mobilization [70], and higher prevalence of subclinical diseases (e.g., lameness, mastitis) [71], all of which exacerbate energy imbalance and increase KR. This underscores that factors beyond milk yield contribute to metabolic vulnerability.

The biological plausibility of higher metabolic risk in first-parity cows and older cows is supported by previous research. This U-shaped parity association has been confirmed in a large-scale German field study on perinatal calf mortality [26], which found the lowest risk in second-parity cows, with higher mortality in both primiparous and older cows. Although focused on reproductive outcomes, these findings support the notion that parity 2 represents an optimal balance between maturity and metabolic capacity, consistent with our KR results.

Another possible explanation, requiring further investigation, for the unexpected non-linearity of KR in our findings, particularly the higher odds observed in cows in the first parity compared to second-parity cows, is the potential under-detection of SCK in primiparous cows. Our approach of identifying KR through milk-based parameters may be more sensitive to subtle metabolic imbalances, thereby revealing cases of poor metabolic adaptation that typically go unnoticed [72]. These subclinical cases may not be routinely tested or diagnosed under standard farm conditions, especially in first-parity cows, who are often assumed to be at lower risk. If future research confirms this hypothesis, it will address a significant knowledge gap, as it is reasonable to assume that undetected SCK contributes not only to economic losses through reduced milk yield but also to compromised health and potential long-term reproductive or metabolic issues.

Based on these findings, we recommend several adjustments to herd health management and propose directions for future research. KR monitoring should be tailored by parity; while multiparous cows are commonly targeted, cows in the first parity also exhibit increased risk and may be under-monitored. Primiparous cows could benefit from better-adapted transition diets and growth-supportive feeding strategies in the prepartum period, highlighting the need for parity-specific management protocols. Despite existing evidence for parity-related differences in metabolic adaption, additional research is required to translate these insights into practical, large-scale risk assessment frameworks. Finally, understanding the mechanisms underlying the relative metabolic resilience observed in second-parity cows could update refined transition protocols for all parity levels.

Notably, there was a significant difference between univariable and multivariable analysis regarding parity. In univariable analysis, primiparous cows had lower odds than multiparous cows (OR 1.37); however, this relationship reversed in the multivariable model. This suggests that confounders, such as breed, housing, or body condition, bias unadjusted estimates, emphasizing the importance of multivariable modeling in epidemiological studies.

### Condition

To make conditions comparable across breeds, BCS was adjusted to the lactation stage and breed beforehand. We found that cows classified as underconditioned exhibited the highest predicted probability of KR (6.4%), with risk decreasing linearly thereafter, reaching the lowest level in overconditioned cows. However, this finding must be interpreted with caution. BCS was assessed at a single point in time, which limits our ability to evaluate prior changes in condition or determine how long KR may have been developing. The condition used in our analysis reflects the cow’s status only at the time of data collection.

Our finding is biologically plausible but leaves the direction of causality uncertain. It does not necessarily contradict the traditional view that overconditioning prepartum is the primary risk factor for ketosis. This established view is supported by extensive research linking excessive body fat reserves before calving to increased fat mobilization and hepatic gluconeogenesis during the transition period, which contributes to metabolic challenges [46]. It is possible that cows previously overconditioned mobilized substantial amounts of body fat reserves due to ongoing (subclinical) ketosis and now appear underconditioned because of disease progression.

This creates a paradox: cows at the highest initial risk (due to overconditioning prepartum) may appear low risk in cross-sectional data if assessed before disease onset, while cows already affected by the disease and its consequences may appear high-risk. The lower KR observed in overconditioned cows may reflect cows that have not yet begun significant fat mobilization, possess better metabolic regulation, thereby delaying the onset of severe NEB, or are in very early stages of KR without observable clinical signs. Consequently, lower KR in overconditioned cows may indicate an earlier point in the metabolic timeline rather than true metabolic resilience. This example also highlights the importance of two-way interactions, as the multivariable results do not account for specific dependencies between predictors; for instance, the interaction between condition and breed is one such case and will be discussed later.

This furthermore highlights a critical limitation of cross-sectional condition assessments. Low condition should not be interpreted as the causal factor for KR, as this could prompt harmful management changes (e.g., discouraging adequate body reserves before calving). Disease-induced weight loss should therefore not be confounded with a predisposing condition. A previous study similarly emphasized that BCS is dynamic and breed-specific; late-lactation or older cows are more likely to be underconditioned due to cumulative metabolic strain, rather than initial underconditioning [36].

### Lameness

Lame cows exhibited 12% higher odds of KR than non-lame cows. Locomotor disorders have long been associated with reduced animal welfare, economic viability, milk yield and reproductive performance [27,61,73–75], and our results show that their role in predicting metabolic imbalances, including KR, is significant, emphasizing the need to consider lameness as both a welfare and metabolic health issue. Lame cows visit the feeding area less frequently than non-lame cows and spend shorter times consuming feed [76].

The elevated risk for ketosis in lame cows likely results from multiple interconnected factors. Studies show that lame cows spend less time feeding and reduce their silage intake even before reaching severe lameness scores, which reduces DMI [77], predisposing them to NEB [7]. NEB is associated with circulating NEFA and BHB, as previously discussed. Furthermore, lameness has been directly linked to decreased milk yield [78], suggesting a systemic metabolic alteration. Another study found that lameness increased milk cortisol levels, a strong indicator of underlying stress [79]. In addition, there is evidence that inflammatory or endocrine stressors (e.g., lipopolysaccharides, in the study’s case) reduce adipose insulin sensitivity and promote lipolysis [80], both of which predispose cows to hyperketonemia.

Our findings have important clinical and management implications. While lameness is widely recognized as a result of housing conditions, on-farm management practices, and the individual animal itself [61], our data suggest that it also contributes to metabolic dysfunction, creating a vicious cycle. Ketosis can induce lethargy, which may worsen lameness. For instance, increased lying time can promote hock lesions, further exacerbating mobility issues [25,61]. It was previously reported that lameness significantly alters resting behavior during the transition period and that such behavioral changes are associated with an elevated risk of ketosis [81]. Our results support and extend this observation, demonstrating that lameness should be integrated into metabolic risk assessment protocols, alongside traditional nutritional and transition cow management factors.

### Two-way interactions

The significance of several risk factors became particularly evident through the results of our two-way interaction analysis. Lactation stage was observed in 6 out of 12 significant interactions, while ECM yield featured prominently in 5, underscoring its central role in influencing KR. This further emphasizes the need to move beyond simple univariable analyses and adopt more nuanced approaches that account for complex interdependencies within the data.

Our first two-way interaction between ECM yield and lactation stage (Fig 3 panel A) illustrates the pronounced association of high ECM yield on KR during early lactation. While cows with low to moderate ECM yield (ECM yield < 35 kg) exhibit an intermediate KR (7.1–12.8%) in the first 99 DIM, high-yielding cows show a markedly elevated risk, 36.0% in early lactation (DIM 0–29) and remaining around 22.6% in mid-early lactation (DIM 30–99). One study has investigated the relationship between milk yield and early lactation in connection with ketosis, finding that initial milk yield during the first weeks postpartum is not only linked to KR but also modulates the reproductive consequences of hyperketonemia [82]. Specifically, cows with higher milk yield experienced fewer negative reproductive consequences at similar BHB levels compared to lower-yielding cows, highlighting how early lactation yield may modulate the subsequent reproductive outcomes of metabolic disease.

The interaction between lactation stage and parity (Fig 3 panel B) offers essential insight. While cows in their second parity and multiparous cows show increased odds of KR during mid-early lactation (DIM 30–99) compared to early lactation (DIM 0–29), first-parity cows exhibit significantly higher odds within the first 29 days postpartum. To our knowledge, no other studies have reported this specific interaction pattern, thereby addressing a notable gap in the current literature. This finding highlights the importance of attentive management during the initial 29 days postpartum for first-parity cows. A physiological explanation for this elevated early-lactation KR in primiparous cows may lie in the abrupt onset of lactation and associated metabolic demands. As the young cow transitions through its first calving and lactation onset, it experiences a combination of hormonal, nutritional, and metabolic adjustments for the first time. Unlike older cows, first-parity cows lack previous physiological adaptation and experience and may have lower metabolic resilience, potentially predisposing them to SCK. This is supported by the idea that primiparous cows often have smaller energy reserves, less efficient feed intake behavior, and ongoing growth requirements, all of which can intensify postpartum energy deficits. This unacknowledged high risk of ketosis in primiparous cows likely stems from the fact that this vulnerability occurs very early in lactation and may coincide with other common postpartum conditions. As clinical attention often shifts toward calving-related diseases or complications during early lactation, SCK in primiparous cows may remain undetected. The complexity of simultaneous stressors during this period can mask metabolic disturbances, increasing the likelihood of underdiagnosis. It is also conceivable that early metabolic challenges in first-parity cows are, to some extent, considered a regular part of first-lactation physiology, highlighting a critical gap in our understanding of how to accurately assess and manage metabolic health in this group during the transition period. This underscores the need for parity-specific health strategies, calling for differentiation in transition cow management and further research into the unique challenges primiparous cows face during their first transition period. Enhanced metabolic monitoring in the first 29 DIM could allow earlier detection and intervention for primiparous cows.

The importance of considering a cow’s breed is highlighted by its interaction with parity (Fig 3 panel C). While GH cows and cows of mixed breed exhibit significantly higher odds of developing KR when multiparous, compared to when they are primiparous or second-parity cows, SIM cattle experience the highest KR during the first parity, with a linear decrease across subsequent lactations. Previous research has shown that SIM cows typically undergo less severe NEB postpartum, manifesting in lower levels of NEFA and ketone bodies compared to purebred GH cows [83]. This may help explain the higher risk of ketosis we observed in SIM cows during their first lactation compared to their GH counterparts, as SIM cows appear to adapt more rapidly in subsequent lactations. In contrast, GH cows demonstrate an increasing susceptibility to KR with advancing parity, consistent with breed-specific physiological adaptations and the previously mentioned association between each 1,000 kg of milk yield in the previous lactation and an increased risk of ketosis in GH cows [18]. Breed-parity dynamics have also been documented: primiparous GH cows seem less prone to ketosis than their counterparts of other breeds (e.g., Jersey cows, in the study’s context), whereas in later parities, the risk becomes more closely associated with milk yield than with breed [56]. These findings highlight not only the significant relevance of breed and parity for KR but also the interaction between them, providing important insights into the multifactorial nature of KR. Multiparous cows, regardless of breed, show comparable odds of KR. This suggests that breed differences in general may be less pronounced in this context.

ECM yield also shows a significant interaction with parity in influencing KR (Fig 3 panel D). While cows with low to average ECM yield have a KR of less than 8% across all parities, high-yielding cows exhibit substantially higher odds in all parity groups, with a particularly elevated risk in primiparous and multiparous cows. This highlights the contribution of high-producing first-parity cows to the increased risk of ketosis observed in parity 1 in our multivariable analysis. While cows producing less than 35 kg ECM yield maintain a consistently low KR regardless of parity, high-yielding cows appear to play a distinctive role in shaping parity-specific risk. This interaction aligns with our findings on the interplay between lactation stage and parity, once again highlighting that primiparous cows differ significantly from older cows, particularly in the context of high-producing demands. These results reinforce our hypothesis that primiparous cows may lack the metabolic resilience needed to meet the energetic challenges of early lactation, as they experience the transition period for the first time. The elevated risk in multiparous cows aligns with our earlier findings, likely reflecting the cumulative demands of successive lactations. Overall, this interaction also highlights the crucial role of milk yield itself and the physiological strain associated with high production, regardless of parity. It suggests a possible threshold relationship, where the odds of KR increase disproportionately beyond a certain ECM yield level, particularly in metabolically unprepared cows. This is why we recommend paying specific attention to management, not only for high-yielding cows in general, but especially for high-yielding primiparous and multiparous cows. This recommendation combines our previously discussed observations on the independent associations of ECM yield and parity with KR. Given the limited availability of studies directly addressing the interplay between ECM yield and parity regarding KR, our findings may offer a novel contribution and highlight the need for further targeted research in this area.

The relationship between breed and ECM yield (Fig 3 panel E) highlights the distinct position of SIM cows. While cows of all breeds show a relatively similar low risk for ketosis at low and average yield, at around 4–7%, and a marked increase in the odds of KR at high yield, high-performing SIM cows exhibit the most pronounced rise in KR. This may suggest that SIM cows reaching very high ECM yield levels may cross a physiological threshold that exceeds their adaptive capacity, particularly during the transition period. Although literature on SIM cows’ specific metabolic response to high ECM yield and its interaction with parity remains limited, two recent studies provide valuable, breed-specific insights. One study highlights differences in milk fat composition in SIM cows based on metabolic health status [84], supporting our observation that high-ECM yield SIM cows may have increased metabolic vulnerability. Another study explores the genetic basis of BHB levels in SIM cows and demonstrates the potential for incorporating ketosis into breeding strategies [85]. These findings support our recommendation for targeted management of high-yielding SIM cows. In our multivariable model, where other predictors were held constant at their mean values, GH cows had lower odds of KR as an individual than SIM at equivalent ECM yield levels. However, because GH cows are more frequently high-yielding, their population-level risk may appear greater when all animals are considered together as a herd or on farm-level. This is consistent with the observation that high-yielding SIM cows show wider confidence intervals, reflecting the smaller number of animals reaching such production levels, while high-yielding GH cows are more common.

The association of the cow’s body condition on KR is significantly shaped by its current lactation stage. The most striking finding in Fig 3 panel F is that underconditioned cows are at particularly high risk for ketosis during mid-early lactation (DIM 30–99), whereas their odds of KR are lower in the immediate postpartum period (DIM 0–29). This risk profile contrasts with that of optimally and overconditioned cows, whose risk remains lower and relatively stable across the first 99 DIM. Importantly, underconditioned cows do not merely start a high risk postpartum, they peak later, suggesting a prolonged or worsening NEB well into mid-early lactation, supported by the sharp increase in their risk trajectory compared with better-fed counterparts. Although literature on this specific stage-condition interaction is scarce, several mechanisms may explain the found pattern. One possibility for later onset of KR in underconditioned cows reflects the duration of undetected SCK earlier in lactation. Since our data capture only a single time point per cow, we cannot track the full progression of disease. It is therefore plausible that some cows experienced persistent SCK, and that prolonged NEB and sustained fat mobilization gradually eroded their body condition, leading to a delayed risk peak, as observed in SIM cows [86]. The key take away is that underconditioned cows in mid-early lactation require particularly close monitoring, even if they appear to have successfully navigated the immediate postpartum transition period. Management strategies should therefore include careful tracking of BCS changes throughout the first 99 DIM, with special attention to post-calving weight loss, to prevent delayed KR onset. Another possibility in management that needs further investigation may be dynamic KR diagnostics, e.g., lowering BHB cut-offs for underconditioned cows around DIM 30–99.

ECM yield modifies the relationship of loose housing with KR (Fig 3 panel G). Most notably, high-yielding cows have significantly lower odds of KR when housed in free-stalls compared with restrictive housing. Nielsen et al. (2023) reported similar subgroup-specific differences in how housing relates to ketosis [67], although comprehensive studies explicitly linking ECM yield, housing and KR are still limited. High-yielding cows have greater energy demands to sustain production, and free-stall housing may support these needs, whether through increased feed availability or enhanced mobility with other health-promoting factors discussed previously. While free-stall housing can also introduce variables such as more difficult monitoring or herd problems due to social or dominance hierarchy, or increased likelihood of lameness, the metabolic advantages for high-yielding cows in this study appear to outweigh these potential drawbacks. Based on these findings, loose housing (e.g., pasture access) should be considered in herd management strategies, particularly when high milk yield is a key breeding and selection objective.

The interaction between ECM yield and body condition (Fig 3 panel H) reveals that the association of body condition on KR is yield-dependent. At low and average yields, body condition is a strong risk factor, as the odds of KR decrease linearly with increasing body condition, from underconditioned (with ca. 8% predicted probability) to normal (ca. 6%) to overconditioned (ca. 4%). However, at high yields this sturdy differentiation diminishes, where all conditions have approximately 12% likelihood of KR. This suggests that when production exceeds 35 kg ECM yield, the metabolic strain associated with sustaining high milk output becomes a dominant factor for KR, potentially counteracting the protective association of optimal or higher body condition. However, when production presents as less extreme, body condition may play a more pronounced modulatory role. Calorimetric assessments in high-yielding cows reveal that NEB is profoundly underestimated by current feeding models and correlates with elevated NEFA and BHB [87], indicative of intense metabolic strain. This underlines the assumption that with high ECM yield, body condition is merely a secondary factor that cannot inhibit the association of yield with KR. Consequently, management should prioritize mitigating NEB and supporting metabolic resilience, specifically in high producing cows, regardless of their body condition.

The interaction between lactation stage and lameness (Fig 3 panel I) underscores their joint association with KR. While no significant difference exists in KR odds between lame and non-lame cows beyond 99 DIM or during the dry period, the first 99 days of lactation present a different pattern. Lame cows not only have overall higher odds of KR but also experience a much steeper increase in risk from early (DIM 0–29) to mid-early lactation (DIM 30–99), whereas cows with good hoof health show only minimal increase. This aligns with biomechanical and metabolic evidence showing that lameness during the dry period can increase KR postpartum, cows that exhibited lameness during dry period were significantly more likely to exceed subclinical BHB thresholds, independent of prepartum lipid mobilization and NEFA [81]. Supporting this, another study found that cows identified as lame within the first 70 DIM had poorer reproductive performance, including delayed pregnancy, indicating that early-lactation lameness disrupts both productivity and health [88]. Our findings add to this evidence by identifying early lactation, particularly DIM 30–99, as the period when lameness most strongly amplifies KR. The stress and reduced mobility associated with lameness likely impair feed intake and disrupt energy homeostasis, increasing vulnerability during the high-demand transition and early lactation phases. The secondary consequences of lameness may accumulate and interact with other stressors of this period, contributing to a peak risk during mid-early lactation. Consequently, management strategies should focus on early detection and intervention for lameness in the dry period and first 99 DIM to enhance metabolic resilience and reduce KR.

KR is significantly associated with the interaction between breed and body condition (Fig 3 panel J). In GH cows, overconditioned cows show the lowest risk and underconditioned cows the highest. Underconditioned and optimally conditioned SIM cows have higher odds than their GH counterparts. In mixed breeds, underconditioned cows have higher odds than SIM or GH. Physiological differences in energy metabolism may help explain these patterns, although detailed research specifically addressing the combination of breed and body condition is scarce. SIM cows exhibit a distinct physiological response to NEB compared to GH counterparts, as reflected in breed-specific NEFA/BHB dynamics and differing susceptibilities to ketosis [54,83]. Genetic variation for milk BHB in SIM cattle further suggests a heritable component to ketosis vulnerability [51]. Cross-breed comparisons confirm that baseline BHB/NEFA levels vary by breed and should be considered when interpreting condition-related risk [11]. Field studies also emphasize that management and feeding systems (e.g., TMR vs. pasture, hoof care routines, housing) modulate these breed-specific associations [6], meaning that breed-BCS interactions should always be interpreted within the context of herd-level factors. Further research should investigate potential differences in DMI between breeds, breed-specific BCS changes during lactation, variation in milk yield, and differences in fat mobilization patterns. Another noteworthy finding is the overall higher risk in the mixed breed category, which may reflect less targeted genetic selection compared with SIM or GH, potentially compromising resilience. Our results also highlight that while underconditioned cows require closer monitoring regardless of breed, optimally conditioned SIM cows merit particular attention, as they exhibit nearly the same predicted probability of KR compared to their underconditioned counterparts. In GH cows, a higher BCS during lactation appears beneficial; however, further research is needed to determine how this interacts with prepartum overconditioning, which is a known predictor of KR.

In restrictively housed cows, the odds of KR are not significantly higher during the first 29 DIM compared with mid lactation (DIM 100–199), but they peak significantly in mid-early lactation (DIM 30–99, Fig 3 panel K). Cows in free-stall housing show a less steep increase in risk from early to mid-early lactation and their KR remains lower than their counterparts during DIM 30–99. Studies examining association of housing and lactation stage remain limited. Future research should focus on the role of housing within the first 99 DIM to clarify the mechanisms behind this interaction. Cows in mid-early lactation may reach production levels where the advantages of loose housing, such as increased voluntary feed intake, improved rumination, and greater mobility, outweigh the benefits of intensive management. Confounding factors such as stress levels, environmental conditions, and differences in physical activity should be considered, as these may also mediate the observed interaction. To better understand the dynamics, future studies could monitor feed intake, metabolic markers (e.g., NEFA, BHB), behavioral patterns, and health outcomes across different housing systems and lactation stages.

Cows of all breeds exhibit higher odds of KR in DIM 30–99 compared to the initial transition phase (DIM 0–29), although this increase is not significant for mixed-breed cows (Fig 3 panel L). SIM cows show the largest increase, starting with the lowest KR among all breeds in DIM 0–29 but reaching the highest risk during DIM 30–99. GH cows show a smaller increase in KR after the first 29 DIM, ultimately displaying the lowest KR among breeds during mid-early lactation. Although studies explicitly examining the interaction between breed and lactation stage in relation to KR are lacking, previously cited research on breed-specific tendencies toward KR can also help contextualize these findings. Breeds may potentially exhibit differences in metabolic adaption speed, milk yield dynamics, and nutrient partitioning, e.g., reaching peak milk yield later, or mobilizing body reserves differently. Additionally, breed-specific differences in feeding behavior, stress resilience, and disease susceptibility may interact with the metabolic demands of each lactation stage. Future research should therefore investigate whether these factors explain the distinct KR patterns of different breed categories in the first 99 DIM.

### Scenarios

The fact that the best-case scenario (average ECM yield (20–35 kg), DIM 200–299, loose housing, not lame, overconditioned, parity 2) is similarly low at 1.5–1.8% across all breeds, and that the worst-case scenario (high ECM yield (>35 kg), DIM 30–99, restrictive housing, lame, underconditioned, first parity) is likewise similar but high at around 35–40% across breeds, suggests that breed-specific susceptibility to KR may be less pronounced when risk factors are highly favorable or unfavorable. This underscores the importance of managing modifiable factors such as lameness, body condition, and housing to mitigate KR across breeds.

Despite the metabolic demands of advanced parity and elevated production, the absence of lameness and adequate body condition contribute to a moderate risk of ketosis in Scenario C (high ECM yield (>35 kg), optimal condition, multiparous, not lame).

The combination of multiple adverse factors, advanced parity, underconditioned, lame, and high production (ECM yield >35 kg), results in a markedly increased risk for Scenario D compared to Scenario C, highlighting the cumulative nature of health and management challenges on KR susceptibility.

While overconditioning may provide some metabolic resilience, the presence of lameness introduces additional stress and disrupts energy balance, resulting in moderate KR in Scenario E (high ECM yield (>35 kg), overconditioned, primiparous, lame). This scenario illustrates how opposing risk factors can contribute to intermediate outcomes and emphasizes the importance of managing both nutrition and mobility in primiparous animals.

Our scenarios illustrate how risk factors can act cumulatively to exacerbate KR susceptibility or, conversely, compensate for one another to yield intermediate outcomes. Such nonlinear dynamics highlight the complexity of disease prediction in dairy herds. From a physiological perspective, these patterns reflect the interplay between energy reserves, nutrient intake, and stress pathways. For example, overconditioned cows can mobilize fat to offset short-term deficits, but this advantage is quickly lost when mobility and health is impaired due to lameness and restrictive housing.

While targeting individual risk factors already offers benefits, the goal should be to address all risk factors collectively rather than in isolation. Although parity and lactation stage are beyond managerial control, it remains essential to optimize controllable factors such as housing, lameness prevention, and body condition. In the longer term, genetic strategies should also be considered, including the prioritization of moderate ECM yields (20–35 kg) rather than maximizing production, as well as the maintenance of uniform breeds within herds to facilitate tailored management.

### Limitations

This study’s cross-sectional design captures the cow’s condition at a single time point, limiting the ability to establish causality or determine the sequence of events. Consequently, the identified relationships should be interpreted as associations, and causal implications must be avoided. Nonetheless, these associations highlight important knowledge gaps and provide a basis for further research to determine definitive causality. Changes in BCS, yield, and health status over the full lactation were not tracked, and some potentially relevant variables, such as BHB blood concentrations, diet composition, feeding behavior, genetics, disease history, calving interval, pasture access and heat stress, were unavailable, leaving room for residual confounding. The dataset may also over- or under-represent certain management systems, or production levels, while broad categories (e.g., “mixed breed,” “loose housing”) may mask important variation. For example, a notable limitation is the numerical imbalance within the housing variable, as the majority of cows were kept in loose housing (95.2% vs. 4.8% in tie-stalls). Although GLMM accounts for such unbalanced data structures by including farm-level variance as a random effect, the smaller sample size in the tie-stall group leads to lower statistical power for this category. Consequently, interaction effects involving housing systems should be interpreted with caution, as the estimates for tie stalls are less precise than those for loose housing. Measurements such as BCS and lameness scoring are susceptible to observer bias, although assessor training aimed to minimize this. Additionally, because concurrent health conditions were not systematically recorded, it was not possible to assess whether the observed increase in odds of KR represented a primary disorder or a secondary condition associated with other disorders (e.g., metritis, mastitis, displaced abomasum). This uncertainty may compromise both the accuracy of KR estimates and the interpretation of some risk-factor associations. For several interactions, limited literature meant that some interpretations are necessarily speculative, underscoring the need for a targeted research approach. It is not possible to determine with certainty that the two-way interactions in our results are the causal drivers of elevated KR or merely an associated factor. Moreover, an important limitation is the absence of blood ketone measurements, which remain the diagnostic gold standard for ketosis. While blood BHB testing provides higher sensitivity and specificity, it is important to note that blood sampling is invasive and often impractical for large-scale, routine monitoring. Thus, its application was not feasible within the scope of this large-scale study involving over 76,000 cows. The use of FPR as a non-invasive proxy is a well-established approach in dairy herd management and epidemiological research, allowing for the analysis of routine data at the population and individual animal level. Consequently, the present analyses focus on KR rather than confirmed clinical disease status. While this distinction should be considered when interpreting the results, the emphasis on risk allows for the identification of animals and management contexts associated with increased susceptibility prior to the onset of metabolic dysfunction. Future research should focus on validating the effectiveness of using FPR-based risk thresholds as a proactive prevention system, specifically evaluating its benefits on long-term herd health and the reduction of clinical ketosis cases.

## Conclusion

This study’s goal was to clarify how production, health and management act, and interact, in relation to KR across different breeds of dairy cows on the individual-animal level. Several novel, stage-dependent, and breed-specific interactions were identified, including heightened vulnerability of high-yielding cows in early lactation, underconditioned cows in mid-early lactation, and SIM cows at high ECM yield or in first lactation.

These results enable veterinarians and herd managers to more accurately assess individual cows, considering breed, lactation stage, body condition, milk yield (ECM yield), housing and health status collectively, rather than evaluating risk-factors in isolation. This integrated approach can improve on-farm decision-making by identifying high-risk animals earlier and guiding preventive or therapeutic actions. Long-term, genetic selection strategies may further balance milk production with resilience, reducing susceptibility over time.

Future research should investigate the physiological mechanisms underlying these patterns to refine detailed, context-dependent KR prevention strategies. In addition, exploring the combination of management interventions with precision monitoring can provide a pathway to tailored, cost-effective approaches across diverse herd systems.

## Supporting information

S1 File Statistical software environment and R packages. Detailed documentation of the R statistical environment (version 4.4.2) and operating system, including a comprehensive list of all used R packages, their specific versions, and full bibliographic references for reproducibility.
(DOCX)

S2 File Extended statistical results for all two-way interactions. This document contains detailed output for the twelve significant two-way interactions, including (1) interaction plots for all factor combinations, (2) tables of Estimated Marginal Means (EMMs) with 95% confidence intervals, and (3) detailed odds ratio (OR) tables for all pairwise comparisons within the interactions (post-hoc tests).
(DOCX)
