# Supplementary material for: Routine milk records reveal novel two-way interactions shaping ketosis risk in German dairy cows
Source: PLoS One. 2026 Jul 16;21(7):e0353380. doi: 10.1371/journal.pone.0353380 (PMC13374898; doi:10.1371/journal.pone.0353380)
Supplement: S1 File — Detailed documentation of the R statistical environment (version 4.4.2) and operating system, including a comprehensive list of all used R packages, their specific versions, and full bibliographic references for reproducibility. (DOCX) [file pone.0353380.s001.docx]

Supplementary Material A

Franziska Gheronte

2026-03-16

SYSTEM:

Analyses were conducted using the R Statistical language (version 4.4.2; R Core Team, 2024) on macOS Sequoia 15.6.1

USED PACKAGES:

- bayesboot (version 0.2.2; Bååth R, 2018)
- lme4 (version 1.1.36; Bates D et al., 2015)
- Matrix (version 1.7.2; Bates D et al., 2025)
- effectsize (version 1.0.0; Ben-Shachar MS et al., 2020)
- DataExplorer (version 0.8.3; Cui B, 2024)
- janitor (version 2.2.1; Firke S, 2024)
- effects (version 4.2.2; Fox J, Weisberg S, 2019)
- carData (version 3.0.5; Fox J et al., 2022)
- flextable (version 0.9.7; Gohel D, Skintzos P, 2024)
- lubridate (version 1.9.4; Grolemund G, Wickham H, 2011)
- ggpubr (version 0.6.0; Kassambara A, 2023)
- robustlmm (version 3.3.1; Koller M, 2016)
- lmerTest (version 3.1.3; Kuznetsova A et al., 2017)
- broom.helpers (version 1.19.0; Larmarange J, Sjoberg D, 2025)
- emmeans (version 1.10.7; Lenth R, 2025)
- randomForest (version 4.7.1.2; Liaw A, Wiener M, 2002)
- sjPlot (version 2.8.17; Lüdecke D, 2024)
- parameters (version 0.24.2; Lüdecke D et al., 2020)
- performance (version 0.13.0; Lüdecke D et al., 2021)
- easystats (version 0.7.4; Lüdecke D et al., 2022)
- see (version 0.10.0; Lüdecke D et al., 2021)
- insight (version 1.1.0; Lüdecke D et al., 2019)
- bayestestR (version 0.15.2; Makowski D et al., 2019)
- modelbased (version 0.9.0; Makowski D et al., 2020)
- report (version 0.6.1; Makowski D et al., 2023)
- correlation (version 0.8.7; Makowski D et al., 2022)
- missRanger (version 2.6.1; Mayer M, 2024)
- tibble (version 3.2.1; Müller K, Wickham H, 2023)
- writexl (version 1.5.1; Ooms J, 2024)
- ggstatsplot (version 0.13.0; Patil I, 2021)
- datawizard (version 1.0.0; Patil I et al., 2022)
- patchwork (version 1.3.0; Pedersen T, 2024)
- R (version 4.4.2; R Core Team, 2024)
- broom (version 1.0.7; Robinson D et al., 2024)
- dlookr (version 0.6.3; Ryu C, 2024)
- hablar (version 0.3.2; Sjoberg D, 2023)
- gtsummary (version 2.1.0; Sjoberg D et al., 2021)
- ggplot2 (version 3.5.1; Wickham H, 2016)
- forcats (version 1.0.0; Wickham H, 2023)
- stringr (version 1.5.1; Wickham H, 2023)
- tidyverse (version 2.0.0; Wickham H et al., 2019)
- readxl (version 1.4.4; Wickham H, Bryan J, 2025)
- dplyr (version 1.1.4; Wickham H et al., 2023)
- purrr (version 1.0.4; Wickham H, Henry L, 2025)
- readr (version 2.1.5; Wickham H et al., 2024)
- tidyr (version 1.3.1; Wickham H et al., 2024)
- knitr (version 1.49; Xie Y, 2024)

PACKAGES CITATION:

- Bååth R (2018). *bayesboot: An Implementation of Rubin’s (1981) Bayesian Bootstrap*. R package version 0.2.2, <https://CRAN.R-project.org/package=bayesboot>.
- Bates D, Mächler M, Bolker B, Walker S (2015). “Fitting Linear Mixed-Effects Models Using lme4.” *Journal of Statistical Software*, *67*(1), 1-48. <doi:10.18637/jss.v067.i01> <https://doi.org/10.18637/jss.v067.i01>.
- Bates D, Maechler M, Jagan M (2025). *Matrix: Sparse and Dense Matrix Classes and Methods*. R package version 1.7-2, <https://CRAN.R-project.org/package=Matrix>.
- Ben-Shachar MS, Lüdecke D, Makowski D (2020). “effectsize: Estimation of Effect Size Indices and Standardized Parameters.” *Journal of Open Source Software*, *5*(56), 2815. <doi:10.21105/joss.02815> <https://doi.org/10.21105/joss.02815>, <https://doi.org/10.21105/joss.02815>.
- Cui B (2024). *DataExplorer: Automate Data Exploration and Treatment*. R package version 0.8.3, <https://CRAN.R-project.org/package=DataExplorer>.
- Firke S (2024). *janitor: Simple Tools for Examining and Cleaning Dirty Data*. R package version 2.2.1, <https://CRAN.R-project.org/package=janitor>.
- Fox J, Weisberg S (2019). *An R Companion to Applied Regression*, 3rd edition. Sage, Thousand Oaks CA. <https://socialsciences.mcmaster.ca/jfox/Books/Companion/index.html>.
- Fox J, Weisberg S (2018). “Visualizing Fit and Lack of Fit in Complex Regression Models with Predictor Effect Plots and Partial Residuals.” *Journal of Statistical Software*, *87*(9), 1-27. <doi:10.18637/jss.v087.i09> <https://doi.org/10.18637/jss.v087.i09>.
- Fox J (2003). “Effect Displays in R for Generalised Linear Models.” *Journal of Statistical Software*, *8*(15), 1-27. <doi:10.18637/jss.v008.i15> <https://doi.org/10.18637/jss.v008.i15>.
- Fox J, Hong J (2009). “Effect Displays in R for Multinomial and Proportional-Odds Logit Models: Extensions to the effects Package.” *Journal of Statistical Software*, *32*(1), 1-24. <doi:10.18637/jss.v032.i01> <https://doi.org/10.18637/jss.v032.i01>.
- Fox J, Weisberg S, Price B (2022). *carData: Companion to Applied Regression Data Sets*. R package version 3.0-5, <https://CRAN.R-project.org/package=carData>.
- Gohel D, Skintzos P (2024). *flextable: Functions for Tabular Reporting*. R package version 0.9.7, <https://CRAN.R-project.org/package=flextable>.
- Grolemund G, Wickham H (2011). “Dates and Times Made Easy with lubridate.” *Journal of Statistical Software*, *40*(3), 1-25. <https://www.jstatsoft.org/v40/i03/>.
- Kassambara A (2023). *ggpubr: ‘ggplot2’ Based Publication Ready Plots*. R package version 0.6.0, <https://CRAN.R-project.org/package=ggpubr>.
- Koller M (2016). “robustlmm: An R Package for Robust Estimation of Linear Mixed-Effects Models.” *Journal of Statistical Software*, *75*(6), 1-24. <doi:10.18637/jss.v075.i06> <https://doi.org/10.18637/jss.v075.i06>.
- Kuznetsova A, Brockhoff PB, Christensen RHB (2017). “lmerTest Package: Tests in Linear Mixed Effects Models.” *Journal of Statistical Software*, *82*(13), 1-26. <doi:10.18637/jss.v082.i13> <https://doi.org/10.18637/jss.v082.i13>.
- Larmarange J, Sjoberg D (2025). *broom.helpers: Helpers for Model Coefficients Tibbles*. R package version 1.19.0, <https://CRAN.R-project.org/package=broom.helpers>.
- Lenth R (2025). *emmeans: Estimated Marginal Means, aka Least-Squares Means*. R package version 1.10.7, <https://CRAN.R-project.org/package=emmeans>.
- Liaw A, Wiener M (2002). “Classification and Regression by randomForest.” *R News*, *2*(3), 18-22. <https://CRAN.R-project.org/doc/Rnews/>.
- Lüdecke D (2024). *sjPlot: Data Visualization for Statistics in Social Science*. R package version 2.8.17, <https://CRAN.R-project.org/package=sjPlot>.
- Lüdecke D, Ben-Shachar M, Patil I, Makowski D (2020). “Extracting, Computing and Exploring the Parameters of Statistical Models using R.” *Journal of Open Source Software*, *5*(53), 2445. <doi:10.21105/joss.02445> <https://doi.org/10.21105/joss.02445>.
- Lüdecke D, Ben-Shachar M, Patil I, Waggoner P, Makowski D (2021). “performance: An R Package for Assessment, Comparison and Testing of Statistical Models.” *Journal of Open Source Software*, *6*(60), 3139. <doi:10.21105/joss.03139> <https://doi.org/10.21105/joss.03139>.
- Lüdecke D, Ben-Shachar M, Patil I, Wiernik B, Bacher E, Thériault R, Makowski D (2022). “easystats: Framework for Easy Statistical Modeling, Visualization, and Reporting.” *CRAN*. <doi:10.32614/CRAN.package.easystats> <https://doi.org/10.32614/CRAN.package.easystats>, R package, <https://easystats.github.io/easystats/>.
- Lüdecke D, Patil I, Ben-Shachar M, Wiernik B, Waggoner P, Makowski D (2021). “see: An R Package for Visualizing Statistical Models.” *Journal of Open Source Software*, *6*(64), 3393. <doi:10.21105/joss.03393> <https://doi.org/10.21105/joss.03393>.
- Lüdecke D, Waggoner P, Makowski D (2019). “insight: A Unified Interface to Access Information from Model Objects in R.” *Journal of Open Source Software*, *4*(38), 1412. <doi:10.21105/joss.01412> <https://doi.org/10.21105/joss.01412>.
- Makowski D, Ben-Shachar M, Lüdecke D (2019). “bayestestR: Describing Effects and their Uncertainty, Existence and Significance within the Bayesian Framework.” *Journal of Open Source Software*, *4*(40), 1541. <doi:10.21105/joss.01541> <https://doi.org/10.21105/joss.01541>, <https://joss.theoj.org/papers/10.21105/joss.01541>.
- Makowski D, Ben-Shachar M, Patil I, Lüdecke D (2020). “Estimation of Model-Based Predictions, Contrasts and Means.” *CRAN*. <https://github.com/easystats/modelbased>.
- Makowski D, Lüdecke D, Patil I, Thériault R, Ben-Shachar M, Wiernik B (2023). “Automated Results Reporting as a Practical Tool to Improve Reproducibility and Methodological Best Practices Adoption.” *CRAN*. <https://easystats.github.io/report/>.
- Makowski D, Wiernik B, Patil I, Lüdecke D, Ben-Shachar M (2022). “correlation: Methods for Correlation Analysis.” Version 0.8.3, <https://CRAN.R-project.org/package=correlation>.
- Makowski D, Ben-Shachar M, Patil I, Lüdecke D (2020). “Methods and Algorithms for Correlation Analysis in R.” *Journal of Open Source Software*, *5*(51), 2306. <doi:10.21105/joss.02306> <https://doi.org/10.21105/joss.02306>, <https://joss.theoj.org/papers/10.21105/joss.02306>.
- Mayer M (2024). *missRanger: Fast Imputation of Missing Values*. R package version 2.6.1, <https://CRAN.R-project.org/package=missRanger>.
- Müller K, Wickham H (2023). *tibble: Simple Data Frames*. R package version 3.2.1, <https://CRAN.R-project.org/package=tibble>.
- Ooms J (2024). *writexl: Export Data Frames to Excel ‘xlsx’ Format*. R package version 1.5.1, <https://CRAN.R-project.org/package=writexl>.
- Patil I (2021). “Visualizations with statistical details: The ‘ggstatsplot’ approach.” *Journal of Open Source Software*, *6*(61), 3167. <doi:10.21105/joss.03167> <https://doi.org/10.21105/joss.03167>, <https://doi.org/10.21105/joss.03167>.
- Patil I, Makowski D, Ben-Shachar M, Wiernik B, Bacher E, Lüdecke D (2022). “datawizard: An R Package for Easy Data Preparation and Statistical Transformations.” *Journal of Open Source Software*, *7*(78), 4684. <doi:10.21105/joss.04684> <https://doi.org/10.21105/joss.04684>.
- Pedersen T (2024). *patchwork: The Composer of Plots*. R package version 1.3.0, <https://CRAN.R-project.org/package=patchwork>.
- R Core Team (2024). *R: A Language and Environment for Statistical Computing*. R Foundation for Statistical Computing, Vienna, Austria. <https://www.R-project.org/>.
- Robinson D, Hayes A, Couch S (2024). *broom: Convert Statistical Objects into Tidy Tibbles*. R package version 1.0.7, <https://CRAN.R-project.org/package=broom>.
- Ryu C (2024). *dlookr: Tools for Data Diagnosis, Exploration, Transformation*. R package version 0.6.3, <https://CRAN.R-project.org/package=dlookr>.
- Sjoberg D (2023). *hablar: Non-Astonishing Results in R*. R package version 0.3.2, <https://CRAN.R-project.org/package=hablar>.
- Sjoberg D, Whiting K, Curry M, Lavery J, Larmarange J (2021). “Reproducible Summary Tables with the gtsummary Package.” *The R Journal*, *13*, 570-580. <doi:10.32614/RJ-2021-053> <https://doi.org/10.32614/RJ-2021-053>, <https://doi.org/10.32614/RJ-2021-053>.
- Wickham H (2016). *ggplot2: Elegant Graphics for Data Analysis*. Springer-Verlag New York. ISBN 978-3-319-24277-4, <https://ggplot2.tidyverse.org>.
- Wickham H (2023). *forcats: Tools for Working with Categorical Variables (Factors)*. R package version 1.0.0, <https://CRAN.R-project.org/package=forcats>.
- Wickham H (2023). *stringr: Simple, Consistent Wrappers for Common String Operations*. R package version 1.5.1, <https://CRAN.R-project.org/package=stringr>.
- Wickham H, Averick M, Bryan J, Chang W, McGowan LD, François R, Grolemund G, Hayes A, Henry L, Hester J, Kuhn M, Pedersen TL, Miller E, Bache SM, Müller K, Ooms J, Robinson D, Seidel DP, Spinu V, Takahashi K, Vaughan D, Wilke C, Woo K, Yutani H (2019). “Welcome to the tidyverse.” *Journal of Open Source Software*, *4*(43), 1686. <doi:10.21105/joss.01686> <https://doi.org/10.21105/joss.01686>.
- Wickham H, Bryan J (2025). *readxl: Read Excel Files*. R package version 1.4.4, <https://CRAN.R-project.org/package=readxl>.
- Wickham H, François R, Henry L, Müller K, Vaughan D (2023). *dplyr: A Grammar of Data Manipulation*. R package version 1.1.4, <https://CRAN.R-project.org/package=dplyr>.
- Wickham H, Henry L (2025). *purrr: Functional Programming Tools*. R package version 1.0.4, <https://CRAN.R-project.org/package=purrr>.
- Wickham H, Hester J, Bryan J (2024). *readr: Read Rectangular Text Data*. R package version 2.1.5, <https://CRAN.R-project.org/package=readr>.
- Wickham H, Vaughan D, Girlich M (2024). *tidyr: Tidy Messy Data*. R package version 1.3.1, <https://CRAN.R-project.org/package=tidyr>.
- Xie Y (2024). *knitr: A General-Purpose Package for Dynamic Report Generation in R*. R package version 1.49, <https://yihui.org/knitr/>.
- Xie Y (2015). *Dynamic Documents with R and knitr*, 2nd edition. Chapman and Hall/CRC, Boca Raton, Florida. ISBN 978-1498716963, <https://yihui.org/knitr/>.
- Xie Y (2014). “knitr: A Comprehensive Tool for Reproducible Research in R.” In Stodden V, Leisch F, Peng RD (eds.), *Implementing Reproducible Computational Research*. Chapman and Hall/CRC. ISBN 978-1466561595.
