# Supplementary material for: Routine milk records reveal novel two-way interactions shaping ketosis risk in German dairy cows
Source: PLoS One. 2026 Jul 16;21(7):e0353380. doi: 10.1371/journal.pone.0353380 (PMC13374898; doi:10.1371/journal.pone.0353380)
Supplement: S2 File — This document contains detailed output for the twelve significant two-way interactions, including (1) interaction plots for all factor combinations, (2) tables of Estimated Marginal Means (EMMs) with 95% confidence intervals, and (3) detailed odds ratio (OR) tables for all pairwise comparisons within the interactions (post-hoc tests). (DOCX) [file pone.0353380.s002.docx]

Supplementary Materials B

Franziska Gheronte

2026-03-16

Inhaltsverzeichnis

[1. Breed / Condition 2](#_Toc219841187)

[2. Breed / Lactation stage 4](#_Toc219841188)

[3. Breed / ECM category 6](#_Toc219841189)

[4. Breed / Parity 8](#_Toc219841190)

[5. Condition / ECM category 10](#_Toc219841191)

[6. Lactation stage / ECM category 12](#_Toc219841192)

[7. Parity / ECM category 15](#_Toc219841193)

[8. Housing / ECM category 18](#_Toc219841194)

[9. Lactation stage / Condition 21](#_Toc219841195)

[10. Lactation stage / Lameness 25](#_Toc219841196)

[11. Lactation stage / Parity 29](#_Toc219841197)

[12. Lactation stage / Housing 33](#_Toc219841198)

# 1. Breed / Condition


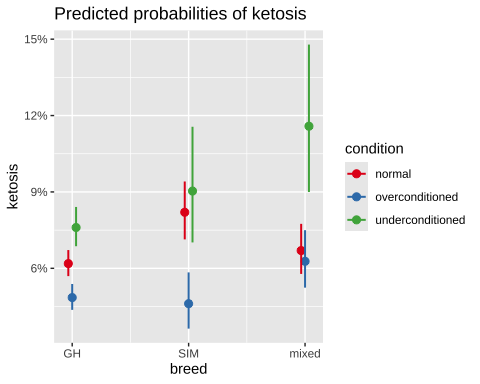


Table S1. Estimated Marginal Means of Ketosis Risk Probability by Breed and Condition.

| **Breed** | **Condition** | **Predicted Probability** | **SE** | **df** | **Lower 95% CI** | **Upper 95% CI** | **null** | **z-ratio** | **p-value** |
| --- | --- | --- | --- | --- | --- | --- | --- | --- | --- |
| GH | normal | 0.062 | 0.003 | Inf | 0.057 | 0.067 | 0.5 | -60.487 | <0.001 |
| SIM | normal | 0.082 | 0.006 | Inf | 0.071 | 0.094 | 0.5 | -31.402 | <0.001 |
| mixed | normal | 0.067 | 0.005 | Inf | 0.058 | 0.077 | 0.5 | -32.886 | <0.001 |
| GH | overconditioned | 0.049 | 0.003 | Inf | 0.044 | 0.054 | 0.5 | -53.080 | <0.001 |
| SIM | overconditioned | 0.046 | 0.006 | Inf | 0.036 | 0.058 | 0.5 | -23.834 | <0.001 |
| mixed | overconditioned | 0.063 | 0.006 | Inf | 0.052 | 0.075 | 0.5 | -27.735 | <0.001 |
| GH | underconditioned | 0.076 | 0.004 | Inf | 0.069 | 0.084 | 0.5 | -44.645 | <0.001 |
| SIM | underconditioned | 0.090 | 0.012 | Inf | 0.070 | 0.116 | 0.5 | -16.482 | <0.001 |
| mixed | underconditioned | 0.116 | 0.015 | Inf | 0.090 | 0.148 | 0.5 | -14.161 | <0.001 |

Table S2. Odds Ratios for Ketosis Risk by Breed within Condition

| **Comparison** | **Condition** | **Odds Ratio** | **Lower 95% CI** | **Upper 95% CI** | **p-value** |
| --- | --- | --- | --- | --- | --- |
| GH / SIM | normal | 0.74 | 0.6 | 0.9 | 0.001 |
| GH / mixed | normal | 0.92 | 0.8 | 1.1 | 0.269 |
| SIM / mixed | normal | 1.24 | 1.0 | 1.6 | 0.062 |
| GH / SIM | overconditioned | 1.05 | 0.8 | 1.5 | 0.699 |
| GH / mixed | overconditioned | 0.76 | 0.6 | 1.0 | 0.017 |
| SIM / mixed | overconditioned | 0.72 | 0.5 | 1.1 | 0.058 |
| GH / SIM | underconditioned | 0.83 | 0.6 | 1.2 | 0.209 |
| GH / mixed | underconditioned | 0.63 | 0.4 | 0.9 | 0.004 |
| SIM / mixed | underconditioned | 0.76 | 0.5 | 1.2 | 0.209 |

# 2. Breed / Lactation stage


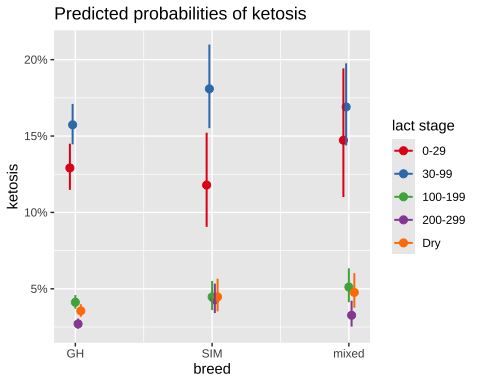


Table S3. Estimated Marginal Means of Ketosis Risk Probability by Breed and Lactation Stage

| **Breed** | **Lactation stage** | **Predicted Probability** | **SE** | **df** | **Lower 95% CI** | **Upper 95% CI** | **null** | **z-ratio** | **p-value** |
| --- | --- | --- | --- | --- | --- | --- | --- | --- | --- |
| GH | 0-29 | 0.129 | 0.008 | Inf | 0.115 | 0.145 | 0.5 | -27.847 | <0.001 |
| SIM | 0-29 | 0.118 | 0.016 | Inf | 0.091 | 0.152 | 0.5 | -13.386 | <0.001 |
| mixed | 0-29 | 0.147 | 0.021 | Inf | 0.110 | 0.194 | 0.5 | -10.312 | <0.001 |
| GH | 30-99 | 0.157 | 0.007 | Inf | 0.145 | 0.171 | 0.5 | -33.008 | <0.001 |
| SIM | 30-99 | 0.181 | 0.014 | Inf | 0.155 | 0.210 | 0.5 | -16.043 | <0.001 |
| mixed | 30-99 | 0.169 | 0.014 | Inf | 0.144 | 0.198 | 0.5 | -16.338 | <0.001 |
| GH | 100-199 | 0.041 | 0.002 | Inf | 0.037 | 0.046 | 0.5 | -54.614 | <0.001 |
| SIM | 100-199 | 0.045 | 0.005 | Inf | 0.036 | 0.055 | 0.5 | -26.995 | <0.001 |
| mixed | 100-199 | 0.051 | 0.006 | Inf | 0.041 | 0.063 | 0.5 | -25.197 | <0.001 |
| GH | 200-299 | 0.027 | 0.002 | Inf | 0.024 | 0.031 | 0.5 | -56.338 | <0.001 |
| SIM | 200-299 | 0.043 | 0.005 | Inf | 0.034 | 0.053 | 0.5 | -26.041 | <0.001 |
| mixed | 200-299 | 0.033 | 0.004 | Inf | 0.025 | 0.042 | 0.5 | -25.035 | <0.001 |
| GH | Dry | 0.036 | 0.002 | Inf | 0.032 | 0.040 | 0.5 | -54.212 | <0.001 |
| SIM | Dry | 0.045 | 0.005 | Inf | 0.035 | 0.057 | 0.5 | -24.155 | <0.001 |
| mixed | Dry | 0.048 | 0.006 | Inf | 0.038 | 0.060 | 0.5 | -23.699 | <0.001 |

Table S4. Odds Ratios for Ketosis Risk by Breed within Lactation Stage

| **Comparison** | **Lactation Stage** | **Odds Ratio** | **Lower 95% CI** | **Upper 95% CI** | **p-value** |
| --- | --- | --- | --- | --- | --- |
| GH / SIM | 0-29 | 1.11 | 0.7 | 1.6 | 0.528 |
| GH / mixed | 0-29 | 0.86 | 0.6 | 1.3 | 0.528 |
| SIM / mixed | 0-29 | 0.77 | 0.5 | 1.3 | 0.528 |
| GH / SIM | 30-99 | 0.85 | 0.7 | 1.1 | 0.328 |
| GH / mixed | 30-99 | 0.92 | 0.7 | 1.2 | 0.534 |
| SIM / mixed | 30-99 | 1.09 | 0.8 | 1.5 | 0.534 |
| GH / SIM | 100-199 | 0.92 | 0.7 | 1.2 | 0.502 |
| GH / mixed | 100-199 | 0.80 | 0.6 | 1.1 | 0.154 |
| SIM / mixed | 100-199 | 0.87 | 0.6 | 1.3 | 0.502 |
| GH / SIM | 200-299 | 0.62 | 0.5 | 0.9 | 0.001 |
| GH / mixed | 200-299 | 0.82 | 0.6 | 1.1 | 0.157 |
| SIM / mixed | 200-299 | 1.32 | 0.9 | 2.0 | 0.157 |
| GH / SIM | Dry | 0.79 | 0.6 | 1.1 | 0.129 |
| GH / mixed | Dry | 0.74 | 0.5 | 1.0 | 0.053 |
| SIM / mixed | Dry | 0.94 | 0.6 | 1.4 | 0.704 |

# 3. Breed / ECM category


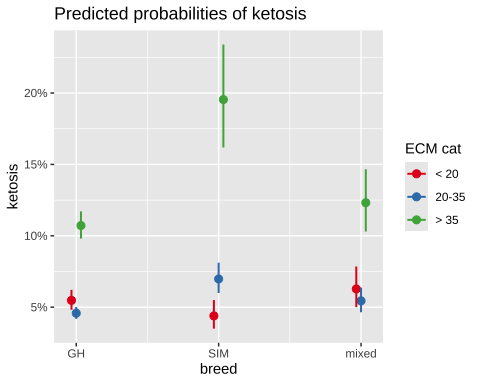


Table S5. Estimated Marginal Means of Ketosis Risk Probability by Breed and ECM Category.

| **Breed** | **ECM category** | **Predicted Probability** | **SE** | **df** | **Lower 95% CI** | **Upper 95% CI** | **null** | **z-ratio** | **p-value** |
| --- | --- | --- | --- | --- | --- | --- | --- | --- | --- |
| GH | < 20 | 0.055 | 0.004 | Inf | 0.048 | 0.062 | 0.5 | -41.681 | <0.001 |
| SIM | < 20 | 0.044 | 0.005 | Inf | 0.035 | 0.055 | 0.5 | -25.530 | <0.001 |
| mixed | < 20 | 0.063 | 0.007 | Inf | 0.050 | 0.079 | 0.5 | -22.038 | <0.001 |
| GH | 20-35 | 0.046 | 0.002 | Inf | 0.042 | 0.050 | 0.5 | -62.285 | <0.001 |
| SIM | 20-35 | 0.070 | 0.005 | Inf | 0.060 | 0.081 | 0.5 | -31.104 | <0.001 |
| mixed | 20-35 | 0.054 | 0.004 | Inf | 0.046 | 0.064 | 0.5 | -33.441 | <0.001 |
| GH | > 35 | 0.107 | 0.005 | Inf | 0.098 | 0.117 | 0.5 | -41.743 | <0.001 |
| SIM | > 35 | 0.195 | 0.018 | Inf | 0.162 | 0.234 | 0.5 | -12.111 | <0.001 |
| mixed | > 35 | 0.123 | 0.011 | Inf | 0.103 | 0.147 | 0.5 | -19.107 | <0.001 |

Table S6. Odds Ratios for Ketosis by Breed within ECM Category

| **Comparison** | **ECM Category** | **Odds Ratio** | **Lower 95% CI** | **Upper 95% CI** | **p-value** |
| --- | --- | --- | --- | --- | --- |
| GH / SIM | < 20 | 1.26 | 0.9 | 1.7 | 0.133 |
| GH / mixed | < 20 | 0.87 | 0.6 | 1.2 | 0.264 |
| SIM / mixed | < 20 | 0.69 | 0.5 | 1.0 | 0.07 |
| GH / SIM | 20-35 | 0.64 | 0.5 | 0.8 | <.001 |
| GH / mixed | 20-35 | 0.83 | 0.7 | 1.0 | 0.026 |
| SIM / mixed | 20-35 | 1.30 | 1.0 | 1.7 | 0.026 |
| GH / SIM | > 35 | 0.49 | 0.4 | 0.7 | <.001 |
| GH / mixed | > 35 | 0.85 | 0.7 | 1.1 | 0.109 |
| SIM / mixed | > 35 | 1.73 | 1.2 | 2.5 | <.001 |

# 4. Breed / Parity


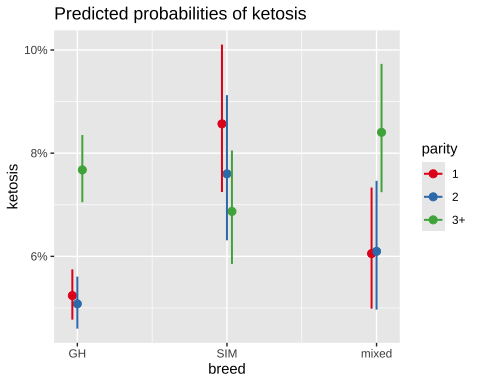


Table S7. Estimated Marginal Means of Ketosis Risk Probability by Breed and Parity

| **Breed** | **Parity** | **Predicted Probability** | **SE** | **df** | **Lower 95% CI** | **Upper 95% CI** | **null** | **z-ratio** | **p-value** |
| --- | --- | --- | --- | --- | --- | --- | --- | --- | --- |
| GH | 1 | 0.052 | 0.002 | Inf | 0.048 | 0.057 | 0.5 | -57.962 | <0.001 |
| SIM | 1 | 0.086 | 0.007 | Inf | 0.072 | 0.101 | 0.5 | -25.517 | <0.001 |
| mixed | 1 | 0.061 | 0.006 | Inf | 0.050 | 0.073 | 0.5 | -26.173 | <0.001 |
| GH | 2 | 0.051 | 0.003 | Inf | 0.046 | 0.056 | 0.5 | -54.944 | <0.001 |
| SIM | 2 | 0.076 | 0.007 | Inf | 0.063 | 0.091 | 0.5 | -24.530 | <0.001 |
| mixed | 2 | 0.061 | 0.006 | Inf | 0.050 | 0.075 | 0.5 | -24.719 | <0.001 |
| GH | 3+ | 0.077 | 0.003 | Inf | 0.071 | 0.084 | 0.5 | -53.126 | <0.001 |
| SIM | 3+ | 0.069 | 0.006 | Inf | 0.059 | 0.081 | 0.5 | -29.796 | <0.001 |
| mixed | 3+ | 0.084 | 0.006 | Inf | 0.072 | 0.097 | 0.5 | -29.055 | <0.001 |

Table S8. Odds Ratios for Ketosis by Breed within Parity

| **Comparison** | **Parity** | **Odds Ratio** | **Lower 95% CI** | **Upper 95% CI** | **p-value** |
| --- | --- | --- | --- | --- | --- |
| GH / SIM | 1 | 0.59 | 0.5 | 0.8 | <.001 |
| GH / mixed | 1 | 0.86 | 0.7 | 1.1 | 0.14 |
| SIM / mixed | 1 | 1.45 | 1.0 | 2.0 | 0.009 |
| GH / SIM | 2 | 0.65 | 0.5 | 0.9 | <.001 |
| GH / mixed | 2 | 0.82 | 0.6 | 1.1 | 0.109 |
| SIM / mixed | 2 | 1.27 | 0.9 | 1.8 | 0.109 |
| GH / SIM | 3+ | 1.13 | 0.9 | 1.4 | 0.22 |
| GH / mixed | 3+ | 0.91 | 0.7 | 1.1 | 0.22 |
| SIM / mixed | 3+ | 0.80 | 0.6 | 1.1 | 0.182 |

# 5. Condition / ECM category


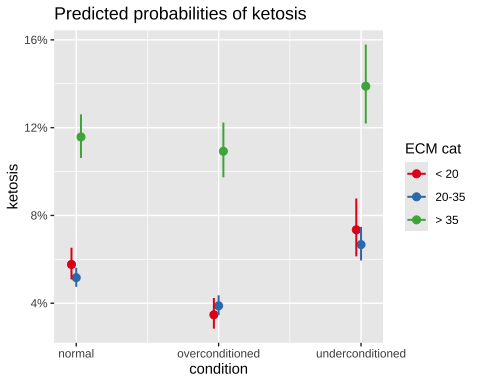


Table S9. Estimated Marginal Means of Ketosis Risk Probability by Condition and ECM Category.

| **Condition** | **ECM category** | **Predicted Probability** | **SE** | **df** | **Lower 95% CI** | **Upper 95% CI** | **null** | **z-ratio** | **p-value** |
| --- | --- | --- | --- | --- | --- | --- | --- | --- | --- |
| normal | < 20 | 0.058 | 0.004 | Inf | 0.051 | 0.065 | 0.5 | -41.326 | <0.001 |
| overconditioned | < 20 | 0.035 | 0.004 | Inf | 0.028 | 0.042 | 0.5 | -31.481 | <0.001 |
| underconditioned | < 20 | 0.073 | 0.007 | Inf | 0.061 | 0.088 | 0.5 | -25.733 | <0.001 |
| normal | 20-35 | 0.052 | 0.002 | Inf | 0.047 | 0.056 | 0.5 | -64.156 | <0.001 |
| overconditioned | 20-35 | 0.039 | 0.002 | Inf | 0.035 | 0.044 | 0.5 | -51.770 | <0.001 |
| underconditioned | 20-35 | 0.067 | 0.004 | Inf | 0.059 | 0.075 | 0.5 | -42.140 | <0.001 |
| normal | > 35 | 0.116 | 0.005 | Inf | 0.106 | 0.126 | 0.5 | -41.138 | <0.001 |
| overconditioned | > 35 | 0.109 | 0.006 | Inf | 0.097 | 0.122 | 0.5 | -32.103 | <0.001 |
| underconditioned | > 35 | 0.139 | 0.009 | Inf | 0.122 | 0.158 | 0.5 | -23.799 | <0.001 |

Table S10. Odds Ratios for Ketosis by Condition within ECM Category

| **Comparison** | **ECM Category** | **Odds Ratio** | **Lower 95% CI** | **Upper 95% CI** | **p-value** |
| --- | --- | --- | --- | --- | --- |
| normal / overconditioned | < 20 | 1.70 | 1.3 | 2.2 | <.001 |
| normal / underconditioned | < 20 | 0.77 | 0.6 | 1.0 | 0.014 |
| overconditioned / underconditioned | < 20 | 0.45 | 0.3 | 0.6 | <.001 |
| normal / overconditioned | 20-35 | 1.35 | 1.2 | 1.5 | <.001 |
| normal / underconditioned | 20-35 | 0.76 | 0.7 | 0.9 | <.001 |
| overconditioned / underconditioned | 20-35 | 0.56 | 0.5 | 0.7 | <.001 |
| normal / overconditioned | > 35 | 1.07 | 0.9 | 1.2 | 0.272 |
| normal / underconditioned | > 35 | 0.81 | 0.7 | 1.0 | 0.006 |
| overconditioned / underconditioned | > 35 | 0.76 | 0.6 | 0.9 | 0.003 |

# 6. Lactation stage / ECM category


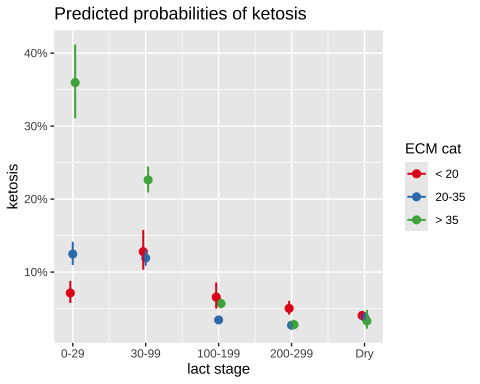


Table S11. Estimated Marginal Means of Ketosis Risk Probability by Lactation Stage and ECM Category.

| **Lactation stage** | **ECM category** | **Predicted Probability** | **SE** | **df** | **Lower 95% CI** | **Upper 95% CI** | **null** | **z-ratio** | **p-value** |
| --- | --- | --- | --- | --- | --- | --- | --- | --- | --- |
| 0-29 | < 20 | 0.071 | 0.008 | Inf | 0.058 | 0.088 | 0.5 | -22.101 | <0.001 |
| 30-99 | < 20 | 0.128 | 0.014 | Inf | 0.103 | 0.158 | 0.5 | -15.470 | <0.001 |
| 100-199 | < 20 | 0.066 | 0.009 | Inf | 0.050 | 0.086 | 0.5 | -18.042 | <0.001 |
| 200-299 | < 20 | 0.050 | 0.005 | Inf | 0.042 | 0.060 | 0.5 | -29.417 | <0.001 |
| Dry | < 20 | 0.040 | 0.003 | Inf | 0.035 | 0.047 | 0.5 | -40.815 | <0.001 |
| 0-29 | 20-35 | 0.125 | 0.008 | Inf | 0.110 | 0.141 | 0.5 | -26.291 | <0.001 |
| 30-99 | 20-35 | 0.119 | 0.006 | Inf | 0.109 | 0.131 | 0.5 | -37.135 | <0.001 |
| 100-199 | 20-35 | 0.034 | 0.002 | Inf | 0.031 | 0.039 | 0.5 | -54.243 | <0.001 |
| 200-299 | 20-35 | 0.027 | 0.002 | Inf | 0.024 | 0.031 | 0.5 | -56.715 | <0.001 |
| Dry | 20-35 | 0.038 | 0.002 | Inf | 0.034 | 0.043 | 0.5 | -52.630 | <0.001 |
| 0-29 | > 35 | 0.360 | 0.026 | Inf | 0.311 | 0.412 | 0.5 | -5.142 | <0.001 |
| 30-99 | > 35 | 0.226 | 0.009 | Inf | 0.209 | 0.245 | 0.5 | -23.785 | <0.001 |
| 100-199 | > 35 | 0.057 | 0.003 | Inf | 0.050 | 0.064 | 0.5 | -43.778 | <0.001 |
| 200-299 | > 35 | 0.028 | 0.003 | Inf | 0.023 | 0.035 | 0.5 | -31.472 | <0.001 |
| Dry | > 35 | 0.033 | 0.006 | Inf | 0.023 | 0.048 | 0.5 | -16.859 | <0.001 |

Table S12. Odds Ratios for Ketosis Risk by Lactation Stage within ECM Category

| **Comparison** | **ECM Category** | **Odds Ratio** | **Lower 95% CI** | **Upper 95% CI** | **p-value** |
| --- | --- | --- | --- | --- | --- |
| (0-29) / (30-99) | < 20 | 0.52 | 0.3 | 0.8 | <.001 |
| (0-29) / (100-199) | < 20 | 1.09 | 0.7 | 1.8 | 0.618 |
| (0-29) / (200-299) | < 20 | 1.45 | 1.0 | 2.2 | 0.013 |
| (0-29) / Dry | < 20 | 1.82 | 1.3 | 2.6 | <.001 |
| (30-99) / (100-199) | < 20 | 2.09 | 1.3 | 3.5 | <.001 |
| (30-99) / (200-299) | < 20 | 2.77 | 1.8 | 4.2 | <.001 |
| (30-99) / Dry | < 20 | 3.49 | 2.4 | 5.1 | <.001 |
| (100-199) / (200-299) | < 20 | 1.33 | 0.8 | 2.1 | 0.098 |
| (100-199) / Dry | < 20 | 1.67 | 1.1 | 2.6 | 0.002 |
| (200-299) / Dry | < 20 | 1.26 | 0.9 | 1.7 | 0.052 |
| (0-29) / (30-99) | 20-35 | 1.05 | 0.9 | 1.3 | 0.483 |
| (0-29) / (100-199) | 20-35 | 4.00 | 3.2 | 5.0 | <.001 |
| (0-29) / (200-299) | 20-35 | 5.10 | 4.1 | 6.4 | <.001 |
| (0-29) / Dry | 20-35 | 3.58 | 2.9 | 4.5 | <.001 |
| (30-99) / (100-199) | 20-35 | 3.80 | 3.2 | 4.5 | <.001 |
| (30-99) / (200-299) | 20-35 | 4.85 | 4.1 | 5.8 | <.001 |
| (30-99) / Dry | 20-35 | 3.40 | 2.9 | 4.0 | <.001 |
| (100-199) / (200-299) | 20-35 | 1.28 | 1.1 | 1.5 | <.001 |
| (100-199) / Dry | 20-35 | 0.89 | 0.7 | 1.1 | 0.105 |
| (200-299) / Dry | 20-35 | 0.70 | 0.6 | 0.8 | <.001 |
| (0-29) / (30-99) | > 35 | 1.92 | 1.4 | 2.6 | <.001 |
| (0-29) / (100-199) | > 35 | 9.32 | 6.7 | 12.9 | <.001 |
| (0-29) / (200-299) | > 35 | 19.55 | 12.9 | 29.6 | <.001 |
| (0-29) / Dry | > 35 | 16.47 | 8.8 | 30.7 | <.001 |
| (30-99) / (100-199) | > 35 | 4.85 | 4.1 | 5.7 | <.001 |
| (30-99) / (200-299) | > 35 | 10.18 | 7.5 | 13.8 | <.001 |
| (30-99) / Dry | > 35 | 8.58 | 4.9 | 15.0 | <.001 |
| (100-199) / (200-299) | > 35 | 2.10 | 1.5 | 2.9 | <.001 |
| (100-199) / Dry | > 35 | 1.77 | 1.0 | 3.1 | 0.005 |
| (200-299) / Dry | > 35 | 0.84 | 0.5 | 1.6 | 0.439 |

# 7. Parity / ECM category


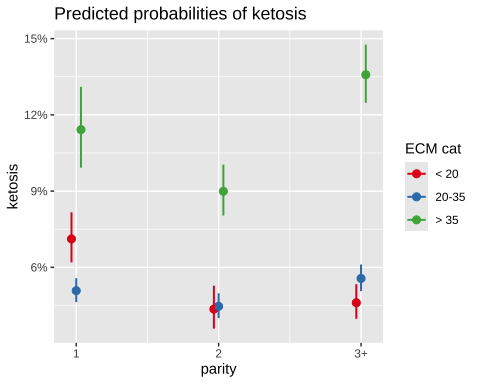


Table S13. Estimated Marginal Means of Ketosis Risk Probability by Parity and ECM Category.

| **Parity** | **ECM category** | **Predicted Probability** | **SE** | **df** | **Lower 95% CI** | **Upper 95% CI** | **null** | **z-ratio** | **p-value** |
| --- | --- | --- | --- | --- | --- | --- | --- | --- | --- |
| 1 | < 20 | 0.071 | 0.005 | Inf | 0.062 | 0.082 | 0.5 | -33.833 | <0.001 |
| 2 | < 20 | 0.044 | 0.004 | Inf | 0.036 | 0.053 | 0.5 | -30.019 | <0.001 |
| 3+ | < 20 | 0.046 | 0.003 | Inf | 0.040 | 0.053 | 0.5 | -38.412 | <0.001 |
| 1 | 20-35 | 0.051 | 0.002 | Inf | 0.046 | 0.056 | 0.5 | -58.764 | <0.001 |
| 2 | 20-35 | 0.045 | 0.002 | Inf | 0.040 | 0.050 | 0.5 | -52.520 | <0.001 |
| 3+ | 20-35 | 0.056 | 0.003 | Inf | 0.051 | 0.061 | 0.5 | -55.721 | <0.001 |
| 1 | > 35 | 0.114 | 0.008 | Inf | 0.099 | 0.131 | 0.5 | -25.595 | <0.001 |
| 2 | > 35 | 0.090 | 0.005 | Inf | 0.080 | 0.100 | 0.5 | -37.153 | <0.001 |
| 3+ | > 35 | 0.136 | 0.006 | Inf | 0.125 | 0.148 | 0.5 | -37.166 | <0.001 |

Table S14. Odds Ratios for Ketosis Risk by Parity within ECM Category

| **Comparison** | **ECM Category** | **Odds Ratio** | **Lower 95% CI** | **Upper 95% CI** | **p-value** |
| --- | --- | --- | --- | --- | --- |
| 1 / 2 | < 20 | 1.68 | 1.3 | 2.2 | <.001 |
| 1 / (3+) | < 20 | 1.59 | 1.3 | 2.0 | <.001 |
| 2 / (3+) | < 20 | 0.94 | 0.7 | 1.2 | 0.614 |
| 1 / 2 | 20-35 | 1.15 | 1.0 | 1.3 | 0.02 |
| 1 / (3+) | 20-35 | 0.91 | 0.8 | 1.0 | 0.042 |
| 2 / (3+) | 20-35 | 0.79 | 0.7 | 0.9 | <.001 |
| 1 / 2 | > 35 | 1.30 | 1.1 | 1.6 | 0.002 |
| 1 / (3+) | > 35 | 0.82 | 0.7 | 1.0 | 0.008 |
| 2 / (3+) | > 35 | 0.63 | 0.5 | 0.7 | <.001 |

# 8. Housing / ECM category


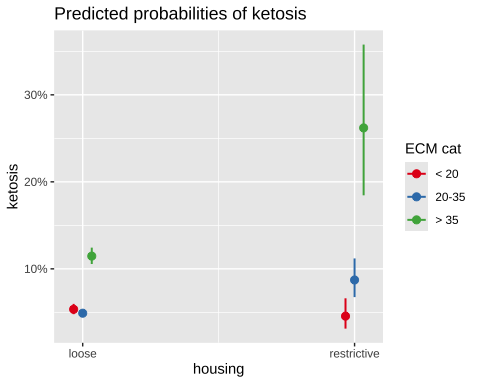


Table S15. Estimated Marginal Means of Ketosis Risk Probability by Housing and ECM Category.

| **Housing** | **ECM category** | **Predicted Probability** | **SE** | **df** | **Lower 95% CI** | **Upper 95% CI** | **null** | **z-ratio** | **p-value** |
| --- | --- | --- | --- | --- | --- | --- | --- | --- | --- |
| loose | < 20 | 0.054 | 0.003 | Inf | 0.048 | 0.060 | 0.5 | -48.282 | <0.001 |
| restrictive | < 20 | 0.046 | 0.009 | Inf | 0.031 | 0.066 | 0.5 | -15.184 | <0.001 |
| loose | 20-35 | 0.049 | 0.002 | Inf | 0.045 | 0.053 | 0.5 | -67.058 | <0.001 |
| restrictive | 20-35 | 0.087 | 0.011 | Inf | 0.068 | 0.112 | 0.5 | -16.597 | <0.001 |
| loose | > 35 | 0.115 | 0.005 | Inf | 0.106 | 0.124 | 0.5 | -43.247 | <0.001 |
| restrictive | > 35 | 0.262 | 0.044 | Inf | 0.185 | 0.358 | 0.5 | -4.506 | <0.001 |

Table S16. Odds Ratios for Ketosis Risk by Housing within ECM Category

| **Comparison** | **ECM Category** | **Odds Ratio** | **Lower 95% CI** | **Upper 95% CI** | **p-value** |
| --- | --- | --- | --- | --- | --- |
| loose / restrictive | < 20 | 1.18 | 0.8 | 1.8 | 0.417 |
| loose / restrictive | 20-35 | 0.54 | 0.4 | 0.7 | <.001 |
| loose / restrictive | > 35 | 0.36 | 0.2 | 0.6 | <.001 |

# 9. Lactation stage / Condition


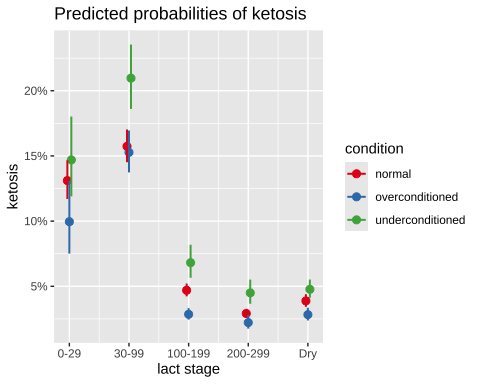


Table S17. Estimated Marginal Means of Ketosis Probability by Lactation Stage and Condition

| **Lactation stage** | **Condition** | **Predicted Probability** | **SE** | **df** | **Lower 95% CI** | **Upper 95% CI** | **null** | **z-ratio** | **p-value** |
| --- | --- | --- | --- | --- | --- | --- | --- | --- | --- |
| 0-29 | normal | 0.131 | 0.008 | Inf | 0.117 | 0.147 | 0.5 | -28.428 | <0.001 |
| 30-99 | normal | 0.157 | 0.006 | Inf | 0.145 | 0.170 | 0.5 | -34.793 | <0.001 |
| 100-199 | normal | 0.047 | 0.003 | Inf | 0.042 | 0.052 | 0.5 | -53.697 | <0.001 |
| 200-299 | normal | 0.029 | 0.002 | Inf | 0.026 | 0.033 | 0.5 | -58.233 | <0.001 |
| Dry | normal | 0.039 | 0.002 | Inf | 0.034 | 0.044 | 0.5 | -48.618 | <0.001 |
| 0-29 | overconditioned | 0.100 | 0.014 | Inf | 0.075 | 0.131 | 0.5 | -13.957 | <0.001 |
| 30-99 | overconditioned | 0.153 | 0.008 | Inf | 0.137 | 0.169 | 0.5 | -27.048 | <0.001 |
| 100-199 | overconditioned | 0.029 | 0.002 | Inf | 0.024 | 0.033 | 0.5 | -43.637 | <0.001 |
| 200-299 | overconditioned | 0.022 | 0.003 | Inf | 0.018 | 0.028 | 0.5 | -31.076 | <0.001 |
| Dry | overconditioned | 0.028 | 0.002 | Inf | 0.024 | 0.034 | 0.5 | -39.123 | <0.001 |
| 0-29 | underconditioned | 0.147 | 0.016 | Inf | 0.119 | 0.180 | 0.5 | -14.155 | <0.001 |
| 30-99 | underconditioned | 0.210 | 0.013 | Inf | 0.186 | 0.235 | 0.5 | -17.460 | <0.001 |
| 100-199 | underconditioned | 0.068 | 0.006 | Inf | 0.057 | 0.082 | 0.5 | -25.824 | <0.001 |
| 200-299 | underconditioned | 0.045 | 0.005 | Inf | 0.037 | 0.055 | 0.5 | -27.888 | <0.001 |
| Dry | underconditioned | 0.048 | 0.004 | Inf | 0.041 | 0.055 | 0.5 | -38.217 | <0.001 |

Table S18. Odds Ratios for Ketosis Risk by Lactation Stage within Condition

| **Comparison** | **Condition** | **Odds Ratio** | **Lower 95% CI** | **Upper 95% CI** | **p-value** |
| --- | --- | --- | --- | --- | --- |
| (0-29) / (30-99) | normal | 0.81 | 0.7 | 1.0 | <.001 |
| (0-29) / (100-199) | normal | 3.06 | 2.5 | 3.7 | <.001 |
| (0-29) / (200-299) | normal | 5.02 | 4.1 | 6.1 | <.001 |
| (0-29) / Dry | normal | 3.74 | 3.0 | 4.6 | <.001 |
| (30-99) / (100-199) | normal | 3.79 | 3.3 | 4.4 | <.001 |
| (30-99) / (200-299) | normal | 6.22 | 5.3 | 7.3 | <.001 |
| (30-99) / Dry | normal | 4.62 | 3.9 | 5.5 | <.001 |
| (100-199) / (200-299) | normal | 1.64 | 1.4 | 2.0 | <.001 |
| (100-199) / Dry | normal | 1.22 | 1.0 | 1.5 | 0.003 |
| (200-299) / Dry | normal | 0.74 | 0.6 | 0.9 | <.001 |
| (0-29) / (30-99) | overconditioned | 0.61 | 0.4 | 1.0 | 0.003 |
| (0-29) / (100-199) | overconditioned | 3.76 | 2.3 | 6.0 | <.001 |
| (0-29) / (200-299) | overconditioned | 4.88 | 2.9 | 8.3 | <.001 |
| (0-29) / Dry | overconditioned | 3.80 | 2.3 | 6.2 | <.001 |
| (30-99) / (100-199) | overconditioned | 6.13 | 4.8 | 7.8 | <.001 |
| (30-99) / (200-299) | overconditioned | 7.95 | 5.6 | 11.3 | <.001 |
| (30-99) / Dry | overconditioned | 6.20 | 4.8 | 8.1 | <.001 |
| (100-199) / (200-299) | overconditioned | 1.30 | 0.9 | 1.9 | 0.068 |
| (100-199) / Dry | overconditioned | 1.01 | 0.7 | 1.4 | 0.923 |
| (200-299) / Dry | overconditioned | 0.78 | 0.5 | 1.2 | 0.084 |
| (0-29) / (30-99) | underconditioned | 0.65 | 0.4 | 0.9 | 0.002 |
| (0-29) / (100-199) | underconditioned | 2.36 | 1.5 | 3.6 | <.001 |
| (0-29) / (200-299) | underconditioned | 3.66 | 2.4 | 5.7 | <.001 |
| (0-29) / Dry | underconditioned | 3.44 | 2.3 | 5.0 | <.001 |
| (30-99) / (100-199) | underconditioned | 3.63 | 2.6 | 5.0 | <.001 |
| (30-99) / (200-299) | underconditioned | 5.63 | 4.0 | 7.9 | <.001 |
| (30-99) / Dry | underconditioned | 5.30 | 4.1 | 6.9 | <.001 |
| (100-199) / (200-299) | underconditioned | 1.55 | 1.1 | 2.3 | 0.002 |
| (100-199) / Dry | underconditioned | 1.46 | 1.1 | 2.0 | 0.002 |
| (200-299) / Dry | underconditioned | 0.94 | 0.7 | 1.3 | 0.617 |

# 10. Lactation stage / Lameness


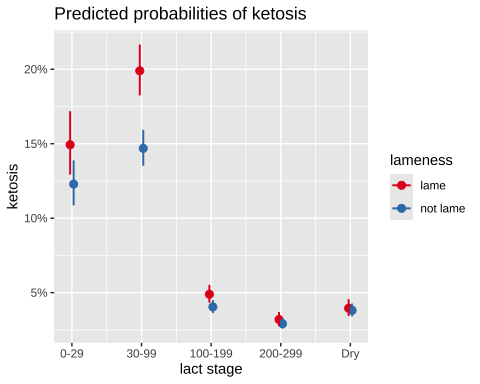


Table S19. Estimated Marginal Means of Ketosis Risk Probability by Lactation Stage and Lameness

| **Lactation stage** | **Lameness** | **Predicted Probability** | **SE** | **df** | **Lower 95% CI** | **Upper 95% CI** | **null** | **z-ratio** | **p-value** |
| --- | --- | --- | --- | --- | --- | --- | --- | --- | --- |
| 0-29 | lame | 0.149 | 0.011 | Inf | 0.129 | 0.172 | 0.5 | -20.387 | <0.001 |
| 30-99 | lame | 0.199 | 0.009 | Inf | 0.182 | 0.217 | 0.5 | -25.517 | <0.001 |
| 100-199 | lame | 0.049 | 0.003 | Inf | 0.043 | 0.055 | 0.5 | -45.339 | <0.001 |
| 200-299 | lame | 0.032 | 0.002 | Inf | 0.028 | 0.037 | 0.5 | -44.255 | <0.001 |
| Dry | lame | 0.040 | 0.003 | Inf | 0.034 | 0.046 | 0.5 | -42.088 | <0.001 |
| 0-29 | not lame | 0.123 | 0.008 | Inf | 0.109 | 0.139 | 0.5 | -27.550 | <0.001 |
| 30-99 | not lame | 0.147 | 0.006 | Inf | 0.135 | 0.159 | 0.5 | -35.909 | <0.001 |
| 100-199 | not lame | 0.040 | 0.002 | Inf | 0.036 | 0.045 | 0.5 | -55.068 | <0.001 |
| 200-299 | not lame | 0.029 | 0.002 | Inf | 0.026 | 0.033 | 0.5 | -55.757 | <0.001 |
| Dry | not lame | 0.038 | 0.002 | Inf | 0.034 | 0.043 | 0.5 | -53.879 | <0.001 |

Table S20. Odds Ratios for Ketosis Risk by Lactation Stage within Lameness

| **Comparison** | **Lameness** | **Odds Ratio** | **Lower 95% CI** | **Upper 95% CI** | **p-value** |
| --- | --- | --- | --- | --- | --- |
| (0-29) / (30-99) | lame | 0.71 | 0.6 | 0.9 | <.001 |
| (0-29) / (100-199) | lame | 3.41 | 2.6 | 4.4 | <.001 |
| (0-29) / (200-299) | lame | 5.32 | 4.0 | 7.1 | <.001 |
| (0-29) / Dry | lame | 4.27 | 3.2 | 5.6 | <.001 |
| (30-99) / (100-199) | lame | 4.83 | 4.0 | 5.8 | <.001 |
| (30-99) / (200-299) | lame | 7.53 | 6.1 | 9.3 | <.001 |
| (30-99) / Dry | lame | 6.04 | 4.9 | 7.5 | <.001 |
| (100-199) / (200-299) | lame | 1.56 | 1.2 | 2.0 | <.001 |
| (100-199) / Dry | lame | 1.25 | 1.0 | 1.6 | 0.008 |
| (200-299) / Dry | lame | 0.80 | 0.6 | 1.0 | 0.017 |
| (0-29) / (30-99) | not lame | 0.81 | 0.7 | 1.0 | 0.003 |
| (0-29) / (100-199) | not lame | 3.33 | 2.7 | 4.1 | <.001 |
| (0-29) / (200-299) | not lame | 4.67 | 3.7 | 5.8 | <.001 |
| (0-29) / Dry | not lame | 3.54 | 2.9 | 4.4 | <.001 |
| (30-99) / (100-199) | not lame | 4.09 | 3.5 | 4.7 | <.001 |
| (30-99) / (200-299) | not lame | 5.74 | 4.9 | 6.8 | <.001 |
| (30-99) / Dry | not lame | 4.35 | 3.7 | 5.1 | <.001 |
| (100-199) / (200-299) | not lame | 1.40 | 1.2 | 1.7 | <.001 |
| (100-199) / Dry | not lame | 1.06 | 0.9 | 1.3 | 0.332 |
| (200-299) / Dry | not lame | 0.76 | 0.6 | 0.9 | <.001 |

# 11. Lactation stage / Parity


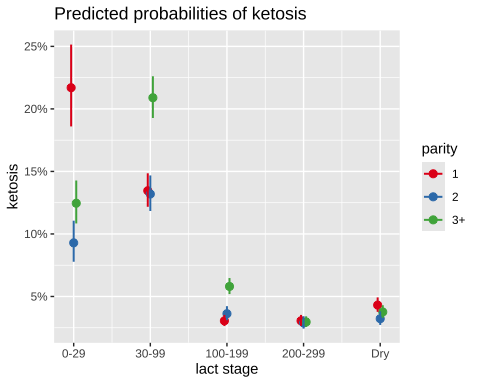


Table S21. Estimated Marginal Means of Ketosis Risk Probability by Lactation Stage and Parity

| **Lactation stage** | **Parity** | **Predicted Probability** | **SE** | **df** | **Lower 95% CI** | **Upper 95% CI** | **null** | **z-ratio** | **p-value** |
| --- | --- | --- | --- | --- | --- | --- | --- | --- | --- |
| 0-29 | 1 | 0.217 | 0.017 | Inf | 0.186 | 0.251 | 0.5 | -13.057 | <0.001 |
| 30-99 | 1 | 0.135 | 0.007 | Inf | 0.122 | 0.149 | 0.5 | -31.749 | <0.001 |
| 100-199 | 1 | 0.031 | 0.002 | Inf | 0.026 | 0.035 | 0.5 | -45.506 | <0.001 |
| 200-299 | 1 | 0.031 | 0.002 | Inf | 0.026 | 0.035 | 0.5 | -45.972 | <0.001 |
| Dry | 1 | 0.043 | 0.003 | Inf | 0.038 | 0.049 | 0.5 | -43.497 | <0.001 |
| 0-29 | 2 | 0.093 | 0.008 | Inf | 0.078 | 0.111 | 0.5 | -22.994 | <0.001 |
| 30-99 | 2 | 0.132 | 0.007 | Inf | 0.118 | 0.147 | 0.5 | -29.765 | <0.001 |
| 100-199 | 2 | 0.036 | 0.003 | Inf | 0.031 | 0.042 | 0.5 | -40.149 | <0.001 |
| 200-299 | 2 | 0.029 | 0.003 | Inf | 0.024 | 0.034 | 0.5 | -39.197 | <0.001 |
| Dry | 2 | 0.032 | 0.003 | Inf | 0.027 | 0.038 | 0.5 | -38.393 | <0.001 |
| 0-29 | 3+ | 0.125 | 0.009 | Inf | 0.108 | 0.143 | 0.5 | -24.311 | <0.001 |
| 30-99 | 3+ | 0.209 | 0.009 | Inf | 0.193 | 0.226 | 0.5 | -25.848 | <0.001 |
| 100-199 | 3+ | 0.058 | 0.003 | Inf | 0.052 | 0.065 | 0.5 | -46.734 | <0.001 |
| 200-299 | 3+ | 0.030 | 0.002 | Inf | 0.026 | 0.034 | 0.5 | -46.613 | <0.001 |
| Dry | 3+ | 0.038 | 0.003 | Inf | 0.033 | 0.043 | 0.5 | -45.478 | <0.001 |

Table S22. Odds Ratios for Ketosis Risk by Lactation Stage within Parity

| **Comparison** | **Parity** | **Odds Ratio** | **Lower 95% CI** | **Upper 95% CI** | **p-value** |
| --- | --- | --- | --- | --- | --- |
| (0-29) / (30-99) | 1 | 1.78 | 1.3 | 2.4 | <.001 |
| (0-29) / (100-199) | 1 | 8.80 | 6.4 | 12.0 | <.001 |
| (0-29) / (200-299) | 1 | 8.78 | 6.4 | 12.0 | <.001 |
| (0-29) / Dry | 1 | 6.15 | 4.5 | 8.3 | <.001 |
| (30-99) / (100-199) | 1 | 4.94 | 4.0 | 6.2 | <.001 |
| (30-99) / (200-299) | 1 | 4.93 | 4.0 | 6.1 | <.001 |
| (30-99) / Dry | 1 | 3.45 | 2.8 | 4.3 | <.001 |
| (100-199) / (200-299) | 1 | 1.00 | 0.8 | 1.3 | 0.988 |
| (100-199) / Dry | 1 | 0.70 | 0.5 | 0.9 | <.001 |
| (200-299) / Dry | 1 | 0.70 | 0.5 | 0.9 | <.001 |
| (0-29) / (30-99) | 2 | 0.67 | 0.5 | 0.9 | <.001 |
| (0-29) / (100-199) | 2 | 2.72 | 2.0 | 3.8 | <.001 |
| (0-29) / (200-299) | 2 | 3.45 | 2.4 | 4.9 | <.001 |
| (0-29) / Dry | 2 | 3.07 | 2.2 | 4.3 | <.001 |
| (30-99) / (100-199) | 2 | 4.04 | 3.2 | 5.2 | <.001 |
| (30-99) / (200-299) | 2 | 5.12 | 3.9 | 6.7 | <.001 |
| (30-99) / Dry | 2 | 4.56 | 3.5 | 5.9 | <.001 |
| (100-199) / (200-299) | 2 | 1.27 | 0.9 | 1.7 | 0.037 |
| (100-199) / Dry | 2 | 1.13 | 0.8 | 1.5 | 0.29 |
| (200-299) / Dry | 2 | 0.89 | 0.6 | 1.2 | 0.311 |
| (0-29) / (30-99) | 3+ | 0.54 | 0.4 | 0.7 | <.001 |
| (0-29) / (100-199) | 3+ | 2.31 | 1.8 | 2.9 | <.001 |
| (0-29) / (200-299) | 3+ | 4.66 | 3.6 | 6.1 | <.001 |
| (0-29) / Dry | 3+ | 3.64 | 2.8 | 4.7 | <.001 |
| (30-99) / (100-199) | 3+ | 4.29 | 3.6 | 5.0 | <.001 |
| (30-99) / (200-299) | 3+ | 8.66 | 7.0 | 10.6 | <.001 |
| (30-99) / Dry | 3+ | 6.76 | 5.6 | 8.2 | <.001 |
| (100-199) / (200-299) | 3+ | 2.02 | 1.6 | 2.5 | <.001 |
| (100-199) / Dry | 3+ | 1.58 | 1.3 | 1.9 | <.001 |
| (200-299) / Dry | 3+ | 0.78 | 0.6 | 1.0 | 0.005 |

# 12. Lactation stage / Housing


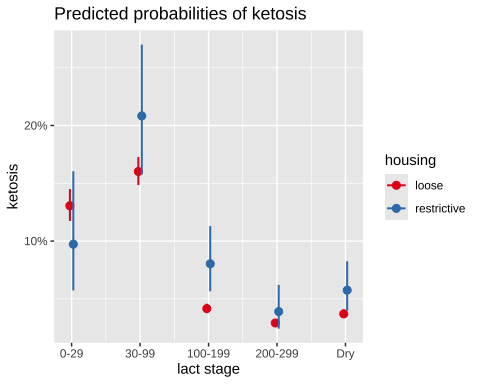


Table S23. Estimated Marginal Means of Ketosis Risk Probability by Lactation Stage and Housing

| **Lactation stage** | **Housing** | **Predicted Probability** | **SE** | **df** | **Lower 95% CI** | **Upper 95% CI** | **null** | **z-ratio** | **p-value** |
| --- | --- | --- | --- | --- | --- | --- | --- | --- | --- |
| 0-29 | loose | 0.131 | 0.007 | Inf | 0.117 | 0.145 | 0.5 | -30.587 | <0.001 |
| 30-99 | loose | 0.160 | 0.006 | Inf | 0.148 | 0.173 | 0.5 | -35.979 | <0.001 |
| 100-199 | loose | 0.042 | 0.002 | Inf | 0.038 | 0.046 | 0.5 | -60.359 | <0.001 |
| 200-299 | loose | 0.029 | 0.002 | Inf | 0.026 | 0.032 | 0.5 | -62.085 | <0.001 |
| Dry | loose | 0.037 | 0.002 | Inf | 0.033 | 0.041 | 0.5 | -59.143 | <0.001 |
| 0-29 | restrictive | 0.097 | 0.026 | Inf | 0.057 | 0.160 | 0.5 | -7.624 | <0.001 |
| 30-99 | restrictive | 0.208 | 0.029 | Inf | 0.158 | 0.270 | 0.5 | -7.683 | <0.001 |
| 100-199 | restrictive | 0.080 | 0.014 | Inf | 0.057 | 0.113 | 0.5 | -12.684 | <0.001 |
| 200-299 | restrictive | 0.039 | 0.009 | Inf | 0.024 | 0.062 | 0.5 | -12.824 | <0.001 |
| Dry | restrictive | 0.058 | 0.011 | Inf | 0.040 | 0.083 | 0.5 | -14.132 | <0.001 |

Table S24. Odds Ratios for Ketosis Risk by Lactation Stage within Housing

| **Comparison** | **Housing** | **Odds Ratio** | **Lower 95% CI** | **Upper 95% CI** | **p-value** |
| --- | --- | --- | --- | --- | --- |
| (0-29) / (30-99) | loose | 0.79 | 0.7 | 0.9 | <.001 |
| (0-29) / (100-199) | loose | 3.46 | 2.9 | 4.1 | <.001 |
| (0-29) / (200-299) | loose | 5.00 | 4.2 | 6.0 | <.001 |
| (0-29) / Dry | loose | 3.90 | 3.3 | 4.6 | <.001 |
| (30-99) / (100-199) | loose | 4.40 | 3.9 | 4.9 | <.001 |
| (30-99) / (200-299) | loose | 6.36 | 5.6 | 7.3 | <.001 |
| (30-99) / Dry | loose | 4.96 | 4.4 | 5.6 | <.001 |
| (100-199) / (200-299) | loose | 1.45 | 1.2 | 1.7 | <.001 |
| (100-199) / Dry | loose | 1.13 | 1.0 | 1.3 | 0.019 |
| (200-299) / Dry | loose | 0.78 | 0.7 | 0.9 | <.001 |
| (0-29) / (30-99) | restrictive | 0.41 | 0.2 | 1.0 | 0.01 |
| (0-29) / (100-199) | restrictive | 1.23 | 0.5 | 3.1 | 0.517 |
| (0-29) / (200-299) | restrictive | 2.66 | 1.0 | 7.4 | 0.012 |
| (0-29) / Dry | restrictive | 1.77 | 0.7 | 4.4 | 0.12 |
| (30-99) / (100-199) | restrictive | 3.01 | 1.6 | 5.5 | <.001 |
| (30-99) / (200-299) | restrictive | 6.48 | 3.0 | 14.0 | <.001 |
| (30-99) / Dry | restrictive | 4.31 | 2.3 | 8.2 | <.001 |
| (100-199) / (200-299) | restrictive | 2.15 | 1.0 | 4.8 | 0.012 |
| (100-199) / Dry | restrictive | 1.43 | 0.7 | 2.8 | 0.174 |
| (200-299) / Dry | restrictive | 0.66 | 0.3 | 1.5 | 0.18 |
