# Supplementary material for: Routine milk records reveal novel two-way interactions shaping ketosis risk in German dairy cows
Source: PLoS One. 2026 Jul 16;21(7):e0353380. doi: 10.1371/journal.pone.0353380 (PMC13374898; doi:10.1371/journal.pone.0353380)
Supplement: Attachment — Submitted filename: Response to Reviewers.docx [file pone.0353380.s004.docx]

Dear Dr. Altay, Dear Reviewers,

Thank you for the opportunity to revise our manuscript „Routine milk records reveal novel two-way interactions shaping ketosis risk in German dairy cows“ (PONE-D-26-13614). We are grateful for the constructive and insightful comments provided by the reviewers.

We have addressed all concerns raised by the Reviewers. A point-by-point response is provided below, and all changes in the manuscript have been highlighted using Track Changes.

Before addressing the comments, we sadly inform the journal that our highly esteemed co-author, Prof. Dr. Kerstin-Elisabeth Müller, passed away during the review process. We have updated the author information and included a dedication in the Acknowledgements to honor her contribution to this work.

In addition to the reviewers’ comments, we have addressed all editorial requirements as follows:

1. Style requirements: We have reviewed the manuscript to ensure it meets PLOS ONE’s style guidelines, including the formatting of the title page, affiliations and file naming.
2. Funding statement: Following the journal’s instructions, we have removed all funding-related information from the Acknowledgements section. We confirm that the funding statement provided in the online submission form is correct and should remain as follows: “Farm visits and data collection for this study were initiated and funded by the German Federal Ministry of Food and Agriculture (BMEL; Bonn, Germany) through the Federal Office for Agriculture and Food (BLE), under grant numbers 2814HS006 (University of Veterinary Medicine Hannover), 2814HS007 (Freie Universität Berlin), and 2814HS008 (Ludwig-Maximilians-Universität Munich). The funders had no role in study design, data collection and analysis, decision to publish, or preparation of the manuscript.”
3. Figure citations: We have ensured that all figures, including the previously mentioned Figure 4.2 (now Fig 2 in the revised numbering), are correctly cited and linked within the text.
4. Figure files: All figures have been removed from the main manuscript file. High-resolution image files have been prepared and uploaded separately as individual files – now with their corrected names.
5. Supporting information: Captions for all Supporting information files (S1 File and S2 File) have been added to the end of the manuscript, and all in-text citations have been updated to match the journal’s guidelines.

Response to Reviewer #1:

| Comment: | Response: |
| --- | --- |
| Conclusions are presented in an appropriate fashion and are supported by the data. The article's content is written in accordance with the journal's guidelines. The article is presented in an intelligible fashion and is written in standard English. | We sincerely thank Reviewer #1 for their time and the positive evaluation of our manuscript. |

Response to Reviewer #2:

| Comment: | Response: |
| --- | --- |
| The manuscript presents a large-scale epidemiological analysis of ketosis risk in German dairy cows using routinely collected milk-record data. The large dataset and the applied statistical approaches represent important strengths of the study. The manuscript is generally well written, clearly structured, and scientifically relevant. The results are biologically plausible and largely consistent with current knowledge regarding ketosis risk factors in dairy cows.  The manuscript would benefit from further clarification and discussion of several methodological aspects that may help strengthen the interpretation of the findings. | We thank Reviewer #2 for the positive and encouraging assessment of our work. We are pleased that the reviewer highlighted the strength of our large-scale dataset and our statistical approach. We also appreciate the constructive criticism regarding the methodological clarifications. We have addressed each of the points raised to further strengthen the scientific rigor and clarity of our manuscript. |
| The primary limitation of the study is the definition of the response variable. The manuscript evaluates “ketosis risk” using milk fat-to-protein ratio (FPR)-based thresholds rather than direct biochemical confirmation (e.g., blood BHBA concentrations). Although the authors appropriately describe the outcome as “risk,” some sections still imply disease occurrence rather than proxy-based metabolic risk. A more detailed discussion of the limitations of FPR as an indirect indicator would strengthen the interpretation of the findings. | We sincerely thank the Reviewer for this important remark. We agree that a precise distinction between a metabolic risk indicator and a clinical diagnosis is crucial for the correct interpretation of our findings and that this should be stated more clearly throughout the manuscript.  The primary objective of our study was not to report clinical disease incidence, but to identify animals at high metabolic risk using a non-invasive, large-scale monitoring tool. We consider this preventive focus a particular strength of the study, as it addresses metabolic imbalances at a stage where proactive management interventions can still prevent the onset of clinical ketosis.  To address the Reviewer’s concern and ensure terminological consistency, we have implemented the following changes:   1. Revised introduction: Lines 78-81. We have added a paragraph explicitly stating that concentrating on risk factor assessment allows for intervention prior to clinical disease onset. We now emphasize that the FPR serves as a proxy for early-stage metabolic dysfunction. 2. Clarification of scope: Lines 122-129. We have clarified that while blood-based metabolite measurements (e.g., BHB) remain the diagnostic gold standard for confirming clinical ketosis, they are often impractical for continuous, individual monitoring in large-scale datasets. Our approach intentionally shifts the focus toward screening and risk assessment. 3. Terminology update: We have carefully screened the manuscript and replaced ambiguous terms or clarified language where needed. We have maintained the term “ketosis” only when referring to general physiological facts or previous studies that explicitly measured the clinical disease. 4. Materials and methods: Line 241f. We have refined our paragraph about FPR to clearly label it as a “non-invasive proxy for metabolic imbalance”. 5. Limitations: Lines 1282-1288. We have also revised the Limitations section. We now clearly explain that BHBA testing is more specific and sensitive but invasive and impractical for large-scale, routine monitoring and also highlight that FPR is used as a non-invasive proxy. We clarify again that we concentrate on ketosis risk rather than confirmed clinical disease status. |
| The manuscript evaluates numerous two-way interactions among predictors. While this is a strength of the study, additional clarification regarding the interaction selection strategy and the issue of multiple testing would improve the statistical interpretation. The possibility of inflated type I error rates resulting from extensive interaction testing could also be discussed. | We appreciate the opportunity to clarify our statistical approach, and we have updated the manuscript accordingly. Foe convenience, the process is summarized below: the selection of predictors and their interactions followed a rigorous, multi-stage process to ensure both clinical relevance and statistical robustness, while minimizing the risk of Type 1 errors (false positives).   1. A-priori clinical selection: Instead of a blind “fishing expedition”, the initial candidate variables were selected a priori based on clinical experience and their established biological relevance in the context of bovine metabolism and the PraeRi colleagues. 2. Multicollinearity check: Multicollinearity among these predictors was formally checked using Variance Inflation Factors (VIF via the car package). Variables causing severe multicollinearity were excluded from simultaneous inclusion to ensure model stability. 3. Univariable GLMM screening: To formally screen the pre-selected candidate variables within a regression framework, univariable generalized mixed-effect models (GLMMs) with a random intercept of farm were constructed. The individual association of each predictor with KR was evaluated using Type II Wald chi-square test (via car::Anova package). Only variables showing a clear contribution to the model (p <0.05 in initial screening) were retained for the multivariable analysis. 4. Pairwise interaction modeling: For the final seven main predictors, we systematically evaluated two-way interactions. To guarantee model stability and allow for a precise estimation of each specific interaction, separate pairwise GLMMs (including the two respective main effects, their interaction term, and the random intercept of farm) were constructed. 5. Addressing Multiple Testing and controlling Type I error: To account for multiple testing during post-hoc pairwise comparisons within each significant interaction effect, p-values were adjusted using the Benjamini-Hochberg (BH) method to control the False Discovery Rate (FDR). We deliberately refrained from applying an additional, global adjustment across the multiple separate interaction models for two reasons: (1) doing so would introduce double-correction, inflating the Type II error rate (missing true biological signals), and (2) since interactions are rarely studied comprehensively in ketosis research, we maintained an explorative approach to avoid masking potentially significant relationships.   We have added a clarifying statement to the Materials and Methods section (Lines 267-286) to explicitly describe this selection strategy and the use of the BH correction. |
| More information regarding missing data and imputation procedures would improve transparency. In particular, the proportion of missing values and the extent of imputation could be reported more clearly. | We fully agree that transparency regarding the handling of missing data is crucial for the reliability of the study’s outcome. We have now more thoroughly expanded the description of our imputation protocol in the Materials and Methods section (Lines 203-215) and would like to highlight the following points here for your convenience:   1. Low proportion of missing data: The extent was very low across all variables, ranging from <0.5 for BCS and milk metabolites to 3.2% for lameness status and 3.37% for parity. Given these small proportions, the risk of introducing significant bias is minimal. 2. Two-stage imputation strategy: We employed a systematic two-stage imputation using the missRanger algorithm. Stage 1 addressed animal-level covariates at the milk-record level, while stage 2 accounted for missing values in parity, breed, body condition and housing data after merging the final dataset. 3. Quality control (OOB Error): To ensure accuracy of the imputed values, we monitored the Out-of-Bag (OOB) error during the Random Forest process (returnOOB = TRUE). This allowed us to verify that the predictive performance of the imputation model was high and that the values were biologically plausible. 4. Avoidance of circularity: We implemented a strict “blinded” approach: the target variables (FPR and ketosis risk) were neither imputed nor used as predictors during the imputation process. They were mathematically derived only after the final imputation was completed, ensuring that no artificial correlations were created between predictors and the metabolic outcome. 5. Reproducibility: All imputations were performed with 1,000 trees and a fixed seed to ensure that the results are fully reproducible.   We believe these additions provide the requested transparency and further support the robustness of our statistical findings. |
| The housing variable is highly imbalanced, with only a small proportion of cows housed in tie stalls. A brief discussion of how this imbalance may affect the interpretation of housing-related estimates and interaction effects would be valuable. | We agree with the Reviewer that the imbalance in housing systems is an important consideration for the interpretation of our model estimates and needs to be addressed as such. We have added a detailed discussion of this limitation in the Discussion section (Lines 1263-1269). We clarified that while the GLMM is robust to unbalanced data, the lower power in the tie-stall group necessitates a cautious interpretation of the corresponding estimates. |
| Because the study is cross-sectional and based on a single time point per animal, associations should be interpreted cautiously, and causal implications should be avoided. | We completely agree with the Reviewer’s assessment. Due to the cross-sectional design of the PraeRi study no definitive causal claims can be made.  To address this, we have taken the following steps:   1. Terminology adjustment: We have carefully reviewed the entire manuscript and ensured that we use non-causal language (e.g., “associations”, “relationships”, or “odds”) rather than terms implying direct causality (e.g., “effects” or “impact”). 2. Expansion of Limitations: We have explicitly stated this limitation in the Discussion section (Lines 1255-1258). We now clarify that while our findings identify important knowledge gaps and provide a robust basis for further research to determine causality, they must be interpreted as associations within the context of this study. |

We would like to thank the reviewers once again for their thoughtful and constructive comments. We believe that the revisions, particularly the expanded discussion of the study’s limitations and the refined statistical descriptions, have significantly improved the clarity and quality of our manuscript.

We hope that these revisions address all the concerns raised and that the manuscript is now suitable for publication in PLOS ONE. We remain available for any further questions or necessary adjustments.

Sincerely,

Franziska Gheronte

(on behalf of all authors)
