# Supplementary material for: Routine milk records reveal novel two-way interactions shaping ketosis risk in German dairy cows
Source: PLoS One. 2026 Jul 16;21(7):e0353380. doi: 10.1371/journal.pone.0353380 (PMC13374898; doi:10.1371/journal.pone.0353380)
Supplement: Peer review history

# Peer review: Decision Letter 0

**DOI:** 10.1371/journal.pone.0353380.r001

**Authors:** Yasin ALTAY

17 May 2026

PONE-D-26-13614

Routine milk records reveal novel two-way interactions shaping ketosis risk in German dairy cows

PLOS One

Dear Dr. Gheronte,

Thank you for submitting your manuscript to PLOS ONE. After careful consideration, we feel that it has merit but does not fully meet PLOS ONE’s publication criteria as it currently stands. Therefore, we invite you to submit a revised version of the manuscript that addresses the points raised during the review process.

==============================

The peer-review process for your paper has now been completed. Following a careful evaluation by two independent expert reviewers, the editorial decision for your manuscript is Major Revision. The reviewers held diverging views regarding the current state of your manuscript. While one reviewer recommended acceptance, the second reviewer raised substantial concerns that necessitate comprehensive revisions. Consequently, to meet the rigorous scientific standards of our journal, it is imperative that the feedback provided by the second reviewer is meticulously considered and thoroughly addressed. We strongly believe that integrating this constructive criticism will significantly enhance the scientific rigor, clarity, and overall impact of your study. When preparing your resubmission, please ensure that you provide a detailed, point-by-point "Response to Reviewers" document. This file must explicitly address each concern raised by the reviewers and clearly outline the corresponding modifications made within the revised text.

==============================

Please submit your revised manuscript by Jul 01 2026 11:59PM. If you will need more time than this to complete your revisions, please reply to this message or contact the journal office at plosone@plos.org. When you're ready to submit your revision, log on to https://www.editorialmanager.com/pone/ and select the 'Submissions Needing Revision' folder to locate your manuscript file.

Please include the following items when submitting your revised manuscript:

- A letter that responds to each point raised by the academic editor and reviewer(s). You should upload this letter as a separate file labeled 'Response to Reviewers'.
- A marked-up copy of your manuscript that highlights changes made to the original version. You should upload this as a separate file labeled 'Revised Manuscript with Track Changes'.
- An unmarked version of your revised paper without tracked changes. You should upload this as a separate file labeled 'Manuscript'.

If you would like to make changes to your financial disclosure, please include your updated statement in your cover letter. Guidelines for resubmitting your figure files are available below the reviewer comments at the end of this letter.

If applicable, we recommend that you deposit your laboratory protocols in protocols.io to enhance the reproducibility of your results. Protocols.io assigns your protocol its own identifier (DOI) so that it can be cited independently in the future. For instructions see: https://journals.plos.org/plosone/s/submission-guidelines#loc-laboratory-protocols. Additionally, PLOS ONE offers an option for publishing peer-reviewed Lab Protocol articles, which describe protocols hosted on protocols.io. Read more information on sharing protocols at https://plos.org/protocols?utm_medium=editorial-email&utm_source=authorletters&utm_campaign=protocols.

As the corresponding author, your ORCID iD is verified in the submission system and will appear in the published article. PLOS supports the use of ORCID, and we encourage all coauthors to register for an ORCID iD and use it as well. Please encourage your coauthors to verify their ORCID iD within the submission system before final acceptance, as unverified ORCID iDs will not appear in the published article. *Only*  the individual author can complete the verification step; PLOS staff *cannot*  verify ORCID iDs on behalf of authors.

We look forward to receiving your revised manuscript.

Kind regards,

Yasin ALTAY, Ph. D.

Academic Editor

PLOS One

Journal Requirements:

When submitting your revision, we need you to address these additional requirements.

1. Please ensure that your manuscript meets PLOS ONE's style requirements, including those for file naming. The PLOS ONE style templates can be found at

https://journals.plos.org/plosone/s/file?id=wjVg/PLOSOne_formatting_sample_main_body.pdf and

https://journals.plos.org/plosone/s/file?id=ba62/PLOSOne_formatting_sample_title_authors_affiliations.pdf

2. Thank you for stating the following in the Acknowledgments Section of your manuscript:

“Farm visits and data collection for this study were initiated and funded by the German Federal Ministry of Food and Agriculture (BMEL; Bonn, Germany) through the Federal Office for Agriculture and Food (BLE), under grant numbers 2814HS006 (University of Veterinary Medicine Hannover), 2814HS007 (Freie Universität Berlin), and 2814HS008 (Ludwig-Maximilians-Universität Munich). The funders had no role in study design, data collection and analysis, decision to publish, or preparation of the manuscript.”

We note that you have provided funding information that is not currently declared in your Funding Statement. However, funding information should not appear in the Acknowledgments section or other areas of your manuscript. We will only publish funding information present in the Funding Statement section of the online submission form.

Please remove any funding-related text from the manuscript and let us know how you would like to update your Funding Statement. Currently, your Funding Statement reads as follows:

“Farm visits and data collection for this study were initiated and funded by the German Federal Ministry of Food and Agriculture (BMEL; Bonn, Germany) through the Federal Office for Agriculture and Food (BLE), under grant numbers 2814HS006 (University of Veterinary Medicine Hannover), 2814HS007 (Freie Universität Berlin), and 2814HS008 (Ludwig-Maximilians-Universität Munich). The funders had no role in study design, data collection and analysis, decision to publish, or preparation of the manuscript.”

Please include your amended statements within your cover letter; we will change the online submission form on your behalf.

3. Please ensure that you refer to Figure 4.2 in your text as, if accepted, production will need this reference to link the reader to the figure.

4. Please remove your figures from within your manuscript file, leaving only the individual TIFF/EPS image files, uploaded separately. These will be automatically included in the reviewers’ PDF.

5. Please include captions for your Supporting Information files at the end of your manuscript, and update any in-text citations to match accordingly. Please see our Supporting Information guidelines for more information: http://journals.plos.org/plosone/s/supporting-information.

6. If the reviewer comments include a recommendation to cite specific previously published works, please review and evaluate these publications to determine whether they are relevant and should be cited. There is no requirement to cite these works unless the editor has indicated otherwise.

[Note: HTML markup is below. Please do not edit.]

Reviewers' comments:

Reviewer's Responses to Questions

**Comments to the Author**

1. Is the manuscript technically sound, and do the data support the conclusions?

The manuscript must describe a technically sound piece of scientific research with data that supports the conclusions. Experiments must have been conducted rigorously, with appropriate controls, replication, and sample sizes. The conclusions must be drawn appropriately based on the data presented.

Reviewer #1: Yes

Reviewer #2: Partly

**********

2. Has the statistical analysis been performed appropriately and rigorously?

Reviewer #1: Yes

Reviewer #2: Yes

**********

3. Have the authors made all data underlying the findings in their manuscript fully available?

The PLOS Data policy requires authors to make all data underlying the findings described in their manuscript fully available without restriction, with rare exception (please refer to the Data Availability Statement in the manuscript PDF file). The data should be provided as part of the manuscript or its supporting information, or deposited to a public repository. For example, in addition to summary statistics, the data points behind means, medians and variance measures should be available. If there are restrictions on publicly sharing data—e.g. participant privacy or use of data from a third party—those must be specified.

Reviewer #1: Yes

Reviewer #2: Yes

**********

4. Is the manuscript presented in an intelligible fashion and written in standard English?

PLOS ONE does not copyedit accepted manuscripts, so the language in submitted articles must be clear, correct, and unambiguous. Any typographical or grammatical errors should be corrected at revision, so please note any specific errors here.

Reviewer #1: Yes

Reviewer #2: Yes

**********

5. Review Comments to the Author

Please use the space provided to explain your answers to the questions above. You may also include additional comments for the author, including concerns about dual publication, research ethics, or publication ethics. (Please upload your review as an attachment if it exceeds 20,000 characters)

Reviewer #1: Conclusions are presented in an appropriate fashion and are supported by the data.

The article's content is written in accordance with the journal's guidelines.

The article is presented in an intelligible fashion and is written in standard English.

Reviewer #2: The manuscript presents a large-scale epidemiological analysis of ketosis risk in German dairy cows using routinely collected milk-record data. The large dataset and the applied statistical approaches represent important strengths of the study. The manuscript is generally well written, clearly structured, and scientifically relevant. The results are biologically plausible and largely consistent with current knowledge regarding ketosis risk factors in dairy cows.

The manuscript would benefit from further clarification and discussion of several methodological aspects that may help strengthen the interpretation of the findings.

Comments:

1. The primary limitation of the study is the definition of the response variable. The manuscript evaluates “ketosis risk” using milk fat-to-protein ratio (FPR)-based thresholds rather than direct biochemical confirmation (e.g., blood BHBA concentrations). Although the authors appropriately describe the outcome as “risk,” some sections still imply disease occurrence rather than proxy-based metabolic risk. A more detailed discussion of the limitations of FPR as an indirect indicator would strengthen the interpretation of the findings.

2. The manuscript evaluates numerous two-way interactions among predictors. While this is a strength of the study, additional clarification regarding the interaction selection strategy and the issue of multiple testing would improve the statistical interpretation. The possibility of inflated type I error rates resulting from extensive interaction testing could also be discussed.

3. More information regarding missing data and imputation procedures would improve transparency. In particular, the proportion of missing values and the extent of imputation could be reported more clearly.

4. The housing variable is highly imbalanced, with only a small proportion of cows housed in tie stalls. A brief discussion of how this imbalance may affect the interpretation of housing-related estimates and interaction effects would be valuable.

5. Because the study is cross-sectional and based on a single time point per animal, associations should be interpreted cautiously, and causal implications should be avoided.

**********

6. PLOS authors have the option to publish the peer review history of their article (what does this mean?). If published, this will include your full peer review and any attached files.

If you choose “no”, your identity will remain anonymous but your review may still be made public.

**Do you want your identity to be public for this peer review?**  For information about this choice, including consent withdrawal, please see our Privacy Policy.

Reviewer #1: No

Reviewer #2: No

**********

[NOTE: If reviewer comments were submitted as an attachment file, they will be attached to this email and accessible via the submission site. Please log into your account, locate the manuscript record, and check for the action link "View Attachments". If this link does not appear, there are no attachment files.]

To ensure your figures meet our technical requirements, please review our figure guidelines: https://journals.plos.org/plosone/s/figures

You may also use PLOS’s free figure tool, NAAS, to help you prepare publication quality figures: https://journals.plos.org/plosone/s/figures#loc-tools-for-figure-preparation.

NAAS will assess whether your figures meet our technical requirements by comparing each figure against our figure specifications.

# Author comment: Author response to Decision Letter 1

**DOI:** 10.1371/journal.pone.0353380.r002

29 May 2026

Dear Dr. Altay,

Thank you very much for the constructive feedback on our manuscript. We have carefully addressed all concerns raised by the reviewers.

Please find our detailed, point-by-point responses to each comment in the separate document labeled 'Response to Reviewers' attached to this submission. The corresponding modifications have been integrated into the revised manuscript (both as a clean version and a version with track changes).

Thank you for your time and for continuing to handle our manuscript.

Kind regards,

Franziska Gheronte

Attachment Submitted filename: Response to Reviewers.docx

# Peer review: Decision Letter 1

**DOI:** 10.1371/journal.pone.0353380.r003

**Authors:** Yasin ALTAY, Yasin ALTAY

23 Jun 2026

Routine milk records reveal novel two-way interactions shaping ketosis risk in German dairy cows

PONE-D-26-13614R1

Dear Dr. Gheronte,

We’re pleased to inform you that your manuscript has been judged scientifically suitable for publication and will be formally accepted for publication once it meets all outstanding technical requirements.

Within one week, you’ll receive an e-mail detailing the required amendments. When these have been addressed, you’ll receive a formal acceptance letter and your manuscript will be scheduled for publication.

An invoice will be generated when your article is formally accepted. Please note, if your institution has a publishing partnership with PLOS and your article meets the relevant criteria, all or part of your publication costs will be covered. Please make sure your user information is up-to-date by logging into Editorial Manager at Editorial Manager® and clicking the ‘Update My Information' link at the top of the page. For questions related to billing, please contact billing support.

If your institution or institutions have a press office, please notify them about your upcoming paper to help maximize its impact. If they’ll be preparing press materials, please inform our press team as soon as possible -- no later than 48 hours after receiving the formal acceptance. Your manuscript will remain under strict press embargo until 2 pm Eastern Time on the date of publication. For more information, please contact onepress@plos.org.

Kind regards,

Yasin ALTAY, Ph. D.

Academic Editor

PLOS One

Additional Editor Comments (optional):

Reviewers' comments:

Reviewer's Responses to Questions

**Comments to the Author**

1. If the authors have adequately addressed your comments raised in a previous round of review and you feel that this manuscript is now acceptable for publication, you may indicate that here to bypass the “Comments to the Author” section, enter your conflict of interest statement in the “Confidential to Editor” section, and submit your "Accept" recommendation.

Reviewer #1: All comments have been addressed

Reviewer #2: All comments have been addressed

**********

2. Is the manuscript technically sound, and do the data support the conclusions?

The manuscript must describe a technically sound piece of scientific research with data that supports the conclusions. Experiments must have been conducted rigorously, with appropriate controls, replication, and sample sizes. The conclusions must be drawn appropriately based on the data presented.

Reviewer #1: Yes

Reviewer #2: Yes

**********

3. Has the statistical analysis been performed appropriately and rigorously?

Reviewer #1: Yes

Reviewer #2: Yes

**********

4. Have the authors made all data underlying the findings in their manuscript fully available?

The PLOS Data policy requires authors to make all data underlying the findings described in their manuscript fully available without restriction, with rare exception (please refer to the Data Availability Statement in the manuscript PDF file). The data should be provided as part of the manuscript or its supporting information, or deposited to a public repository. For example, in addition to summary statistics, the data points behind means, medians and variance measures should be available. If there are restrictions on publicly sharing data—e.g. participant privacy or use of data from a third party—those must be specified.

Reviewer #1: Yes

Reviewer #2: Yes

**********

5. Is the manuscript presented in an intelligible fashion and written in standard English?

PLOS ONE does not copyedit accepted manuscripts, so the language in submitted articles must be clear, correct, and unambiguous. Any typographical or grammatical errors should be corrected at revision, so please note any specific errors here.

Reviewer #1: Yes

Reviewer #2: Yes

**********

6. Review Comments to the Author

Please use the space provided to explain your answers to the questions above. You may also include additional comments for the author, including concerns about dual publication, research ethics, or publication ethics. (Please upload your review as an attachment if it exceeds 20,000 characters)

Reviewer #1: The corrections given for the study entitled "Routine milk records reveal novel bidirectional interactions shaping the risk of ketosis in German dairy cows" have been taken into consideration. The article can be accepted in its current form.

Reviewer #2: The authors have adequately addressed my previous comments. The manuscript has improved and is now acceptable for publication. I only suggest checking minor typographical errors and the consistency of the reported housing proportions during final editing.

**********

7. PLOS authors have the option to publish the peer review history of their article (what does this mean?). If published, this will include your full peer review and any attached files.

If you choose “no”, your identity will remain anonymous but your review may still be made public.

**Do you want your identity to be public for this peer review?**  For information about this choice, including consent withdrawal, please see our Privacy Policy.

Reviewer #1: No

Reviewer #2: No

**********

# Editor report: Acceptance letter

**DOI:** 10.1371/journal.pone.0353380.r004

**Authors:** Yasin ALTAY, Yasin ALTAY

PONE-D-26-13614R1

PLOS One

Dear Dr. Gheronte,

I'm pleased to inform you that your manuscript has been deemed suitable for publication in PLOS One. Congratulations! Your manuscript is now being handed over to our production team.

At this stage, our production department will prepare your paper for publication. This includes ensuring the following:

* All references, tables, and figures are properly cited

* All relevant supporting information is included in the manuscript submission,

* There are no issues that prevent the paper from being properly typeset

You will receive further instructions from the production team, including instructions on how to review your proof when it is ready. Please keep in mind that we are working through a large volume of accepted articles, so please give us a few days to review your paper and let you know the next and final steps.

Lastly, if your institution or institutions have a press office, please let them know about your upcoming paper now to help maximize its impact. If they'll be preparing press materials, please inform our press team within the next 48 hours. Your manuscript will remain under strict press embargo until 2 pm Eastern Time on the date of publication. For more information, please contact onepress@plos.org.

You will receive an invoice from PLOS for your publication fee after your manuscript has reached the completed accept phase. If you receive an email requesting payment before acceptance or for any other service, this may be a phishing scheme. Learn how to identify phishing emails and protect your accounts at https://explore.plos.org/phishing.

If we can help with anything else, please email us at customercare@plos.org.

Thank you for submitting your work to PLOS ONE and supporting open access.

Kind regards,

PLOS ONE Editorial Office Staff

on behalf of

Dr Yasin ALTAY

Academic Editor

PLOS One
